# HDAC7-Induced Epigenetic Repression Modulates ATF3 Functional Plasticity in Colorectal Cancer Pathogenesis

**DOI:** 10.7150/ijbs.121348

**Published:** 2026-05-01

**Authors:** Qi Wang, Donglei Ji, Yanjie Jia, Shuang Li, Wenjing Gao, Tingting Liang, Yingying Liang, Caroline Zeng, Chunyu Wang, Ka Lung Cheung, Quan Wang, Ming-Ming Zhou, Lei Zeng

**Affiliations:** 1 Bethune Institute of Epigenetic Medicine, The First Hospital of Jilin University, Changchun, Jilin, 130021, China.; 2 International Center of Future Science, Jilin University, Changchun, 130012, China.; 3 Department of Pathology, The First Hospital of Jilin University, Changchun, China.; 4 Department of Gastrointestinal Surgery, The First Hospital of Jilin University, Changchun, 130021, China.; 5 Pennsylvania State University College of Medicine, Hershey, PA 17033, USA.; 6 State Key Laboratory of Supramolecular Structure and Materials, College of Chemistry, Jilin University, Changchun, Jilin, 130012, China.; 7 Department of Pharmacological Sciences, Icahn School of Medicine at Mount Sinai, New York, NY, 10029, USA.

**Keywords:** colorectal cancer, HDAC7, ATF3, class IIa HDAC, epigenetic repression, scaffold protein

## Abstract

Class IIa histone deacetylase 7 (HDAC7) regulates transcription primarily through scaffolding functions, but its molecular mechanisms in cancer pathogenesis remain incompletely understood. Here, we establish HDAC7 as a key epigenetic regulator in colorectal cancer (CRC). HDAC7 is overexpressed in CRC tumors and correlates with advanced disease stages, lymph node metastasis, and poor patient survival. Mechanistically, HDAC7 scaffolds a repressive complex with HDAC3 and the stress-responsive transcription factor ATF3. This reduces H3K27ac/H3K18ac occupancy and blocks BRD4/RNA polymerase II (Pol II) recruitment at *ATF3* regulatory regions to epigenetically silence its transcription. Consequently, this repression inactivates ATF3's tumor-suppressive functions, activating oncogenic PI3K-Akt signaling while suppressing the Hippo pathway. Genetic depletion or pharmacological inhibition of HDAC7 disrupts this repressive complex, triggering a functional switch in ATF3. This promotes BRD4/Pol II recruitment and H3K27ac enrichment at the ATF3 locus, enabling ATF3 to undergo transcriptional self-activation. Reactivated ATF3 suppresses CRC proliferation and survival by downregulating Bcl-2, upregulating p21 (*CDKN1A*) to induce cell cycle arrest, promoting caspase-3-mediated apoptosis, and inhibiting PI3K-Akt signaling. Xenograft studies confirm that HDAC7 depletion suppresses tumorigenicity *in vivo*. Our work identifies HDAC7 as a molecular mediator that governs ATF3's functional plasticity through competitive cofactor recruitment, positioning HDAC7 inhibition as a therapeutic strategy to reactivate ATF3-mediated tumor suppression in CRC.

## Introduction

Colorectal cancer (CRC) is the third most commonly diagnosed cancer and the second leading cause of cancer-related deaths worldwide, posing a major clinical challenge 1. Despite advances in diagnosis and treatment, both the incidence and mortality of CRC continue to rise, underscoring an urgent need for novel therapeutic strategies 2. While epigenetic dysregulation, notably aberrant histone acetylation, is a well-established driver of CRC pathogenesis 3, the exact mechanisms and key regulators involved remain incompletely understood. Identifying pivotal epigenetic regulators and their molecular interactions is critical for developing targeted therapies to address the escalating burden of CRC.

The balance of histone acetylation, dynamically controlled by histone acetyltransferases (HATs) and histone deacetylases (HDACs), is a fundamental regulator of gene expression 4. Generally, histone hyperacetylation relaxes chromatin to activate transcription, often of oncogenes, whereas hypoacetylation compacts chromatin to silence tumor suppressors 5. Among the 18 HDAC members categorized into four major classes 6, the Class IIa subfamily (HDAC4, 5, 7, 9) is notable for low intrinsic deacetylase activity and tissue-specific regulatory functions 7. HDAC7 is unique because it requires association with the SMRT/N-CoR-HDAC3 complex for full enzymatic activity 8 and exhibits context-dependent roles in cancer. For example, HDAC7 overexpression can suppress c-MYC and induce apoptosis in some hematologic malignancies like pro-B acute lymphoblastic leukemia and Burkitt lymphoma cells 9. In contrast, its knockdown downregulates c-MYC by upregulating tumor suppressors p21^Cip1^ and p27^Kip1^ in cervical (HeLa), breast (MCF7), and colon (HCT-116) solid tumor cell lines 10. Notably, HDAC7 is overexpressed in CRC tissues and positively correlates with advanced disease 11, yet the specific mechanism of HDAC7 in CRC pathogenesis remains insufficiently understood.

Activating transcription factor-3 (ATF3), a stress-inducible bZIP transcription factor within the ATF/CREB family, further complicates this picture through its functional duality in cancer. ATF3 binds CRE motifs (5'-TGACGTCA-3') to exert context-dependent functions in stress responses and cancer progression 12. Although basally expressed at low levels, ATF3 is robustly induced under stress 13, forming homo- or hetero-dimers that regulate transcription through promoter and enhancer binding 14. ATF3 regulates a diverse network of pathways, including PI3K-Akt 15, NF-κB 16, Toll-like receptor 4 signaling 17, actin cytoskeleton dynamics 18, transforming growth factor-β (TGF-β) 19, and p53 signaling 20. Additionally, ATF3 directly controls key apoptosis and cell-cycle regulators such as Bcl-2, p21 and cyclins 21-23, and modulates cell death mechanisms, notably switching from apoptosis to necroptosis in the hepatic steatosis condition 24. In CRC, ATF3 is downregulated in tumors compared to adjacent normal tissues and can function as a tumor suppressor 25. Its loss enhances cell migration and survival 26, while its overexpression induces apoptosis via Bcl-2 suppression and PARP cleavage 27, 28. Paradoxically, in certain CRC contexts (e.g., HT29 and Caco-2 cells), ATF3 overexpression appears to promote invasion and motility 29. This context-dependent switching is stage-dependent, influenced by tumor microenvironment 30 and variable expression levels across CRC lines 31, suggesting that ATF3's activity is determined by upstream epigenetic mechanisms.

Supporting this epigenetic mode of regulation, ATF3 expression can be reactivated by HDAC inhibitors, leading to apoptosis 32. Class I HDACs, such as HDAC3, are known to form repressive complexes at the ATF3-bound promoter, suppressing both ATF3 expression and its downstream target genes 33. Furthermore, ATF3-binding sites overlap with regions of active chromatin marked by H3K27ac and the co-activator p300 34, implying that its transcriptional output is determined by a competitive balance between co-activators and co-repressors. However, whether Class IIa HDACs, particularly HDAC7, participate in this competitive regulation to modulate ATF3's functional switch in CRC remains uncharacterized.

In this study, we identify HDAC7 as an epigenetic regulator that controls ATF3's functional plasticity in CRC. We demonstrate that HDAC7 is overexpressed in aggressive CRC and correlates with poor patient survival. Mechanistically, HDAC7 functions as an oncogenic scaffold, which recruits HDAC3 and ATF3 into a repressive complex at the *ATF3* locus independent of its catalytic domain. This complex silences ATF3 transcription by reducing H3K27ac and excluding co-activators like BRD4 and Pol II. Genetic depletion or chemical inhibition of HDAC7 disrupts this repressive complex, enabling ATF3 to recruit co-activators and activate its own expression. Reactivated ATF3 then implements a tumor-suppressive program through Bcl-2 downregulation, p21 upregulation, and apoptosis induction. Our work defines an HDAC7-dependent epigenetic modulation that controls ATF3's dual roles, from a repressor state (bound to HDAC3) to an activator state (complexed with BRD4/Pol II), highlighting HDAC7 inhibition as a therapeutic strategy to reactivate ATF3-mediated tumor suppression in CRC.

## Methods

### Human colon tissues collection and ethics statements

Eighty paired colorectal tissue specimens (tumor and adjacent normal tissues) were collected from treatment-naïve CRC patients undergoing curative resection at the First Hospital of Jilin University between 2019 and 2020. Patients had received no prior neoadjuvant chemotherapy or radiotherapy. Immediately following resection (within 5 minutes), tumor and normal tissues were collected, snap-frozen in liquid nitrogen, and transferred to a -80 °C refrigerator for long-term storage prior to RNA and protein extraction. Comprehensive clinicopathological data, including patient age, gender, tumor location, and TNM staging were recorded for subsequent analysis. For comparative studies, the CRC patients in **Table 1** were stratified into high and low HDAC7 expression groups based on the median HDAC7 expression level in adjacent normal tissues. All procedures were conducted in accordance with the principles of the Declaration of Helsinki (2013). Written informed consent was obtained from all patients, and the study protocol was approved by the Institute Research Ethics Committee of the First Hospital of Jilin University.

### Cell culture and reagents

All human CRC cell lines (SW480, SW620, LS174T, HCT-116, RKO, HT29, Caco-2, NCI-H508) and HEK293T cells were cultured in media supplemented with 10% heat-inactivated fetal bovine serum (FBS; Gibco, 16000-044), 50 U/mL penicillin, and 50 µg/mL streptomycin (Gibco, 15140). The specific media conditions were: SW480, SW620, HCT-116, HT29, and HEK293T in Dulbecco's modified Eagle's medium (DMEM) (Sigma, D6429); LS174T and Caco-2 in RPMI-1640 (Gibco, 11875); RKO and NCI-H508 in McCoy's 5A (Gibco, 16600). Cells were incubated at 37 °C in a humidified atmosphere with 5% CO_2_ (Thermo Scientific Heracell 150i), and the culture medium was refreshed every 48 h. For plasmid transfection, Polyethyleneimine Linear (PEI; Polysciences, 23966) was used, while siRNA transfections were performed using EL Transfection Reagent (TransIntro, FT201-02). Pharmacological inhibitors, including Vorinostat (SAHA; pan-HDAC inhibitor), TMP269 (class IIa HDAC inhibitor), and JQ1 (BET inhibitor), were purchased from Selleck Chemicals (Munich, Germany). Stock solutions (10 mM in DMSO) were prepared and stored at -80 °C. For inhibitor treatments, cells were seeded and allowed to adhere for 24 h before being incubated with the indicated concentrations of SAHA, TMP269, or JQ1 for durations of 2, 8, 12, 24, or 48 h. The final DMSO concentration in the treatment medium did not exceed 0.05% (v/v). Control cells were treated with an equivalent volume of DMSO.

### Plasmid construction

Full-length human *HDAC7* (NM_001258401.2) and *ATF3* (NM_001030287.4) coding sequences (CDS) were amplified by PCR from HEK293T cDNA using Q5 High-Fidelity DNA Polymerase. *HDAC7* fragments were cloned into the pcDNA3.1-Flag vector (Invitrogen, V79020) via *EcoRI* and *XhoI* restriction sites to generate N-terminal Flag-tagged constructs. *ATF3* was cloned into the pEGFP-C3 (Clontech, 6082-1) using *KpnI* and *EcoRI* sites to create a C-terminal GFP fusion construct. All final constructs were verified by bidirectional Sanger sequencing (Comate Bioscience). Plasmids were purified using the GeneJET Plasmid Midiprep Kits (Thermo Fisher Scientific, USA, K210015) and stored at -80 °C in TE buffer (10 mM Tris-HCl, 1 mM EDTA, pH 8.0).

### Establishment of stable HDAC7-knockdown cell lines

Stable HDAC7-knockdown cell lines were generated through lentiviral transduction. Lentiviral particles were generated by co-transfecting HEK293T cells with the pLKO1-shHDAC7 plasmid (target sequence: 5'-GATCCGGGTGCACAGTAAATA-3'; Sigma USA, TRCN0000255687) and the packaging plasmids (pAX and pMD2.G). Virus-containing supernatant was collected 24 or 48 h after transfection, filtered, and used to infect the CRC cell lines SW480, SW620, HT29, HCT-116, and LS174T. A non-targeting shRNA (pLKO.1-shNC) was used as a control. Infected cells were selected with puromycin (2 µg/mL) for 3 weeks to obtain stable polyclonal populations. Knockdown efficiency was confirmed by Western blotting and qRT**-**PCR.

### Histopathology and immunohistochemistry

For histopathological analysis, the segregated CRC tumor and matched adjacent normal tissue samples were fixed in 10% neutral-buffered formalin (Sigma, HT501128) at room temperature. Following fixation, the tissues were processed, embedded in paraffin, and sectioned into 5-μm-thick slices. Sections were then deparaffinized and rehydrated for staining. For histomorphology assessment, sections were stained with Hematoxylin and Eosin (H&E) and with periodic acid-Schiff (PAS) according to standard protocols. Immunohistochemistry (IHC) was performed on paraffin-embedded tissue according to standard practice, using the primary antibodies against HDAC7 (Abcam, Cambridge, UK, #ab166911, 1:2000) and anti-Ki67 (Proteintech, Rosemont, IL, #27309-1-AP, 1:10000).

### Cell proliferation assay

EdU Incorporation Assay: Cell proliferation was assessed using the 5-ethynyl-2'-deoxyuridine (EdU) Cell Proliferation Kit. Colorectal cancer cells with stable shRNA-mediated HDAC7 knockdown and scramble control (Scr) were seeded in 12-well plates at a density of 5×10^4^ cells per well. After being cultured for 24 h, cells were treated with EdU at a final concentration of 10 μM and incubated for 2 h. Cells were fixed with 3.7% paraformaldehyde for 15 min, then washed three times with PBS, and permeabilized with 0.5% Triton X-100 in PBS (1 mL per well) for 10 min at room temperature. The Click-iT reaction mixture was applied for 30 min, followed by nuclear staining with Hoechst 33342 for 25 min in the dark. After three additional PBS washes, cells were observed and imaged using an Olympus microscope (Japan). The percentage of EdU-positive cells was quantified using ImageJ software (NIH, Bethesda, MD) and normalized to the total number of Hoechst-stained nuclei.

MTT viability assay: Cells were seeded in 96-well plates at a density of 2-4×10^3^ cells per well with three technical replicates per condition. After 16 h, cells were treated with various concentrations of SAHA (pan-HDAC inhibitor) or TMP269 (class IIa HDAC inhibitor) for 24, 48, and 72 h. Subsequently, MTT solution (3-[4,5-dimethylthiazol-2-yl]-2,5-diphenyl tetrazolium bromide) (5 mg/mL; Sigma Aldrich, USA) was added to each well (20 µL per well) and incubated for 4 h. The medium was carefully removed, and the resulting formazan crystals were solubilized with 200 μL DMSO. Absorbance was measured at 570 nm using a Multiskan FC microplate reader (Thermo Fisher Scientific, USA). Data were analyzed using GraphPad Prism 8.0 software (GraphPad Software, San Diego, CA, USA).

### Colony formation assay

For clonogenic assay analysis, 200-2000 cells were seeded per well in 6-well plates and cultured for 10-14 days to allow colony formation. Once visible colonies consisting of > 50 cells had formed, cells were fixed with 3.7% paraformaldehyde and stained with 0.1% crystal violet dye (Sigma-Aldrich, USA). Stained colonies were imaged and counted using ImageJ software (NIH, Bethesda, MD).

### Cell cycle analysis and apoptosis assays by flow cytometry

Cell cycle distribution and apoptosis were analyzed by flow cytometry in SW480 and SW620 cells under two experimental conditions: (1) stable HDAC7 knockdown versus scramble control (Scr) cells, and (2) cells treated for 48 h with 1.5 μM SAHA, 3 μM TMP269, or an equivalent volume of DMSO (vehicle control).

For cell cycle analysis, cells were harvested by trypsinization, washed three times with ice-cold PBS, and fixed in 70% ethanol overnight at 4 °C. After fixation, cells were washed with PBS and stained with propidium iodide (PI) solution (50 μg/mL) containing RNase A. Cell cycle phase distribution was analyzed using a FACS Calibur flow cytometer (BD Biosciences, USA), and data were quantified with FlowJo VX software (Tree Star Inc., Ashland, OR).

Apoptosis was assessed using an Annexin V-EGFP/PI Apoptosis Detection Kit (Yeasen Biotechnology, Shanghai, China) according to the manufacturer's instructions. Briefly, cells were collected, washed with cold PBS, and resuspended in 1× binding buffer. Annexin V-EGFP and PI were sequentially added, and samples were incubated in the dark for 15 min at room temperature. Apoptotic cells were immediately analyzed by flow cytometry. A minimum of 10,000 events were recorded per sample, and the percentages of early apoptotic (Annexin V+/PI-) and late apoptotic (Annexin V+/PI+) cells were determined using FlowJo VX software (Tree Star Inc., Ashland, OR).

### Transwell migration assay

Cell migration was assessed using 8-μm pore Transwell inserts (Corning, Bedford, MA, Cat: 3422). Stable shRNA-mediated HDAC7 knockdown or control cells were seeded at 8×10^4^ cells per insert into the upper chamber in serum-free DMEM. For inhibitor treatments, cells were pre-incubated for 48 h with 1.5 μM SAHA, 3 μM TMP269, or 0.05% DMSO (vehicle control) prior to seeding. The lower chamber contained DMEM supplemented with 20% FBS as a chemoattractant. After 48 h of incubation, non-migrated cells on the upper membrane surface were removed with a cotton swab. Migrated cells on the lower membrane surface were fixed with 3.7% paraformaldehyde for 30 min, stained with 0.1% crystal violet for 15 min, and imaged under a light microscope. The number of migrated cells was quantified in five random fields per insert using ImageJ software (NIH, Bethesda, MD). Each experiment was performed in at least three independent replicates.

### siRNA-mediated gene knockdown

Gene knockdown was performed using siRNA transfection in SW480 cells. Cells were seeded in 6-well plates at a density of 2×10^5^ cells per well and allowed to adhere overnight. The following siRNA duplexes (purchased from Genepharma) were used for transfection:

Target Gene siRNA Name Sequence (5'-3')

ATF3 ATF3-1

F: GAGGCGACGAGAAAGAAAUTT

R: AUUUCUUUCUCGUCGCCUCTT

ATF3 ATF3-2

F: GAUGAGAGAAACCUCUUUATT

R: UAAAGAGGUUUCUCUCAUCTT

HDAC7 HDAC7-1

F: CAACCUGAAGCUGCGCUAUAATT

R: UUAUAGCGCAGCUUCAGGUUGTT

HDAC7 HDAC7-2

F: GAUCCGGGUGCACAGUAAAUATT

R: UAUUUACUGUGCACCCGGAUCTT

HDAC3 HDAC3-1

F: CAAGAGUCUUAAUGCCUUCAATT

R: UUGAAGGCAUUAAGACUCUUGTT

HDAC3 HDAC3-2

F: GCACCCAAUGAGUUCUAUGAUTT

R: AUCAUAGAACUCAUUGGGUGCTT

siRNA duplexes were diluted in serum-free transfection medium and mixed with DharmaFECT#1 transfection reagent (Thermo Fisher). The siRNA-lipid complexes were incubated for 20 min at room temperature before being added to the cells. After 24 h of transfection, cells were harvested for RNA extraction. Knockdown efficiency was assessed by quantitative PCR (qPCR) analysis, with target gene mRNA expression levels normalized to appropriate housekeeping controls.

### RNA isolation, cDNA synthesis, and qRT-PCR

Total RNA was extracted from tissue samples or cultured cells using Trizol reagent (Invitrogen; Thermo Fisher Scientific) according to the manufacturer's instructions. RNA was reverse-transcribed into cDNA using the Hifair® II 1st Strand cDNA Synthesis Kit (Yeasen Biotechnology, Shanghai, China). Quantitative real-time PCR (qRT-PCR) was performed with Maxima SYBR Green/Rox qPCR Master Mix (Thermo Scientific, USA) and gene-specific primers (sequences provided in Supplementary Table 1). Amplification conditions followed the manufacturer's thermal cycling protocol. Gene expression levels were calculated using the 2-ΔΔCt method and normalized to *GAPDH* as an internal control. Each experiment was performed in duplicate and carried out in at least three independent biological replicates.

### RNA sequencing

RNA sequencing (RNA-seq) was performed on SW480 cells stably expressing shHDAC7 or scramble control (Scr) shRNA. The sequencing service was conducted by Beijing Genomics Institute (BGI, Shenzhen, China) using the BGISEQ or DNBSEQ platform according to their standard protocols. Total RNA was extracted, and library preparation and paired-end sequencing were carried out using BGI's proprietary kit. Three technical replicates were included per group to ensure reproducibility. Raw data processing, including quality control, read alignment, and gene expression quantification, was performed by BGI. Subsequent bioinformatic analysis (differential gene expression and functional enrichment) was conducted using the Dr. Tom online platform (http://report.bgi.com).

### Western blot analysis

Total protein was extracted from cells or tissues using RIPA lysis buffer (Thermo Fisher Scientific) supplemented with protease and phosphatase inhibitor cocktails. Protein concentrations were determined using a BCA Protein Quantification Kit (Yeasen, Shanghai, China). Equal amounts of protein were separated by SDS-PAGE on 8%, 10%, or 12% gels and transferred to PVDF membranes. The membranes were blocked with 8% skim milk in TBST for 1 h at room temperature and then incubated with primary antibodies overnight at 4°C with gentle shaking. The following primary antibodies were used: Rabbit monoclonal anti-HDAC7 (Abcam, Cambridge, UK, #ab166911, 1:2000; Biolegend, San Diego, CA, #683502, 1:800; Santa Cruz, Dallas, TX, #sc-47563, 1:600), Rabbit monoclonal anti-ATF3 (Abcam, Cambridge, UK) (#ab207434, 1:1500), Rabbit monoclonal anti-PI 3 Kinase catalytic subunit gamma/PI3K-gamma (Abcam, Cambridge, UK) (#ab133595, 1:2000), Rabbit polyclonal anti-Phospho-PI3K p85 (Tyr458) (Tyr467)/p55 (Tyr199) (Affinity, Cincinnati, OH) (#AF3242, 1:800), Akt (pan) Rabbit mAb (Cell Signal Tech, Danvers, MA) (#4691, 1:2000), Phospho-Akt (Ser473) XP® Rabbit mAb (Cell Signal Tech, Danvers, MA) (#4060, 1:1000), Cleaved Caspase-3 (Asp175) antibody (Cell Signal Tech, Danvers, MA) (#9661T, 1:800), Caspase-8/p43/p18 Polyclonal (Proteintech, Rosemont, IL) (#13423-1-ap, 1:800), Rabbit monoclonal anti-Bcl-2 (Abcam, Cambridge, UK) (#ab182858, 1:2000), Cyclin D1 Polyclonal antibody (Proteintech, Rosemont, IL) (#26939-1-AP, 1:800), Rabbit monoclonal anti-GAPDH (Abcam, Cambridge, UK) (#ab181602, 1:20000), HDAC3 (D2O1K) Rabbit mAb (Cell Signal Tech, Danvers, MA) (#85057, 1:2000), p21 Waf1/Cip1 (12D1) Rabbit mAb (Cell Signal Tech, Danvers, MA) (#2947S, 1:2000), mouse monoclonal anti-p53 (Abcam, Cambridge, UK) (#ab1101, 1:2000), Rabbit Polyclonal anti-acetyl-p53 (Lys373, Lys382) (Merck Millipore, Burlington, MA) (#06-785, 1:2000), rabbit monoclonal anti-YAP1 (Abcam, Cambridge, UK) (#ab52771, 1:10000), Phospho-YAP1 (Ser127) Rabbit pAb (Zen-Bioscience, Chengdu, SC) (#381297, 1:500), Src (36D10) Rabbit mAb (Cell Signal Tech, Danvers, MA) (#2109, 1:2000), Phospho-Src Family (Tyr416) (D49G4) Rabbit mAb (Cell Signal Tech, Danvers, MA) (#6943, 1:2000). After washing with TBST, membranes were incubated with horseradish peroxidase (HRP)-conjugated anti-rabbit or anti-mouse secondary antibodies (Beyotime, Shanghai, China) for 1 h at room temperature. Protein bands were visualized using an ECL detection kit (Merck Millipore) and imaged with a chemiluminescence system.

### Chromatin immunoprecipitation (ChIP) assay

ChIP assays were performed in SW480 cells with stable HDAC7 knockdown (shHDAC7) or scramble control (Scr). Cells were crosslinked with 1% formaldehyde for 10 min at room temperature. The reaction was quenched with 125 mM glycine for 5 min. After washing three times with cold PBS, cells were lysed in ChIP lysis buffer and sonicated to generate chromatin fragments of 200-500 bp. Immunoprecipitation was carried out overnight at 4 °C with the following antibodies: anti-H3K27ac (Abcam, #ab4729, 1:200), anti-H3K18ac (Abcam, #ab40888, 1:200), anti-ATF3 (Abcam, #ab207434, 2 μg), anti-BRD4 (Abcam, #ab128874, 1:200), and anti-RNA polymerase II Ser2P/Ser5P (Cell Signaling Technology, #13546S, 1:200). Normal mouse IgG (Santa Cruz, #2025, 1:200) and normal rabbit IgG (Cell Signaling Technology, #2729, 1:200) were used as negative controls. Immunoprecipitated DNA was purified using a QIAquick Spin Kit (Qiagen) and analyzed by qPCR with primers targeting the *ATF3* enhancer or promoter regions (see **Supplementary Table 2** for sequences).

### Co-Immunoprecipitation (Co-IP) assay

Co-immunoprecipitation assays were performed in HEK293T cells co-transfected with plasmids encoding Flag-HDAC7 (or HDAC7-targeting siRNA) and GFP-ATF3. Cells were lysed in IP lysis buffer (25 mM Tris pH 7.4, 150 mM NaCl, 1 mM EDTA, 1% NP-40, and 5% glycerol). Lysates were centrifuged, and the supernatants were incubated overnight at 4 °C with rotation in the presence of the following antibodies: anti-HDAC7 (Abcam, #ab166911, 1:2000), anti-HDAC3 (Cell Signaling Technology, #85057, 1:2000), anti-BRD4 (Abcam, #ab128874, 1:2000), anti-GFP (Engibody, #AT0028, 1:2000; or Bioss, #bs-0639R, 1:500), or anti-FLAG M2 (Sigma-Aldrich, #F1804, 1:2000). Normal rabbit IgG (Cell Signaling Technology, #2729, 1:2000) was used as a negative control. Protein A/G magnetic beads (Invitrogen) were then added, and the mixtures were incubated for 2 h at 4 °C. Beads were washed six times with lysis buffer to remove unbound proteins. Bound proteins were eluted in 2× SDS sample buffer and analyzed by SDS-PAGE and Western blotting.

### Luciferase reporter transcription assay

Transcriptional activity of the *ATF3* promoter was investigated using a dual-luciferase reporter system. The pGL3-ATF3 plasmid (Promega, E1761), containing a 1.5-kb human *ATF3* promoter fragment (-1486 to +114 relative to TSS), was co-transfected with the pRL-TK normalization vector (Promega, E2241) in a 10:1 ratio using Lipofectamine 3000. Firefly and Renilla luciferase activities were measured sequentially using the Dual-Glo Luciferase Assay System (Promega, E2920) on a GloMax Navigator luminometer. Relative promoter activity was calculated as the ratio of Firefly to *Renilla* luciferase activity, expressed as relative light units (RLU), and normalized to both empty pGL3-basic control and vehicle-treated samples, with background subtraction using lysis buffer-only blank signals. Data are from five biological replicates, each with three technical replicates.

### Animal experiments

All animal procedures were approved by the Experimental Animal Ethical Committee of Jilin University (#A2021-028) and conducted in compliance with the institutional guidelines. Twenty female BALB/c nude mice (approximately 4 weeks old, 16-18 g) were purchased from SPF Biotechnology (Beijing, China) and acclimatized for 7 days under specific pathogen-free (SPF) conditions (22 ± 1 °C, 55 ± 5% humidity, 12 h light/dark cycle). Mice were stratified by weight and randomized into two groups (n = 10 per group). SW480 cells stably expressing shHDAC7 or scramble control (Scr) shRNA were harvested, resuspended in PBS, and subcutaneously injected into the left flank of each mouse (1×10^6^ cells in 100 μL, 27G needle) under isoflurane anesthesia. Mice that exhibited injection failure or in which tumors failed to engraft (tumor volume < 50 mm^3^ on day 7) were excluded from the study. Tumor dimensions were measured three times weekly by using digital calipers, and volumes were calculated as (Length×Width^2^)/2. Humane endpoints were defined as tumor volume exceeding 1500 mm^3^ or weight loss > 20%. After 28 days, all mice were euthanized by CO_2_ asphyxiation (30% chamber volume displacement per minute). Tumors were excised, weighed (Sartorius, CPA225D), and processed for snap-freezing or fixation for subsequent analysis.

### Statistical analysis

Statistical analyses were performed using GraphPad Prism 8 (v9.3.1; GraphPad Software Inc., La Jolla, CA) or SPSS (v28.0). For multi-group comparisons, one-way analysis of variance (ANOVA) was employed. Differences between samples and their respective control groups were evaluated using either unpaired or paired two-tailed Student's *t*-test. Survival outcomes were analyzed using Kaplan-Meier curves, with significance determined by the log-rank test. Data are presented as mean ± standard deviation (SD) or standard error of the mean (SEM), based on at least three independent experiments. A *p*-value < 0.05 was considered statistically significant.

## Results

### HDAC7 overexpression correlates with advanced disease and reduced survival in CRC

**Figure 1:**
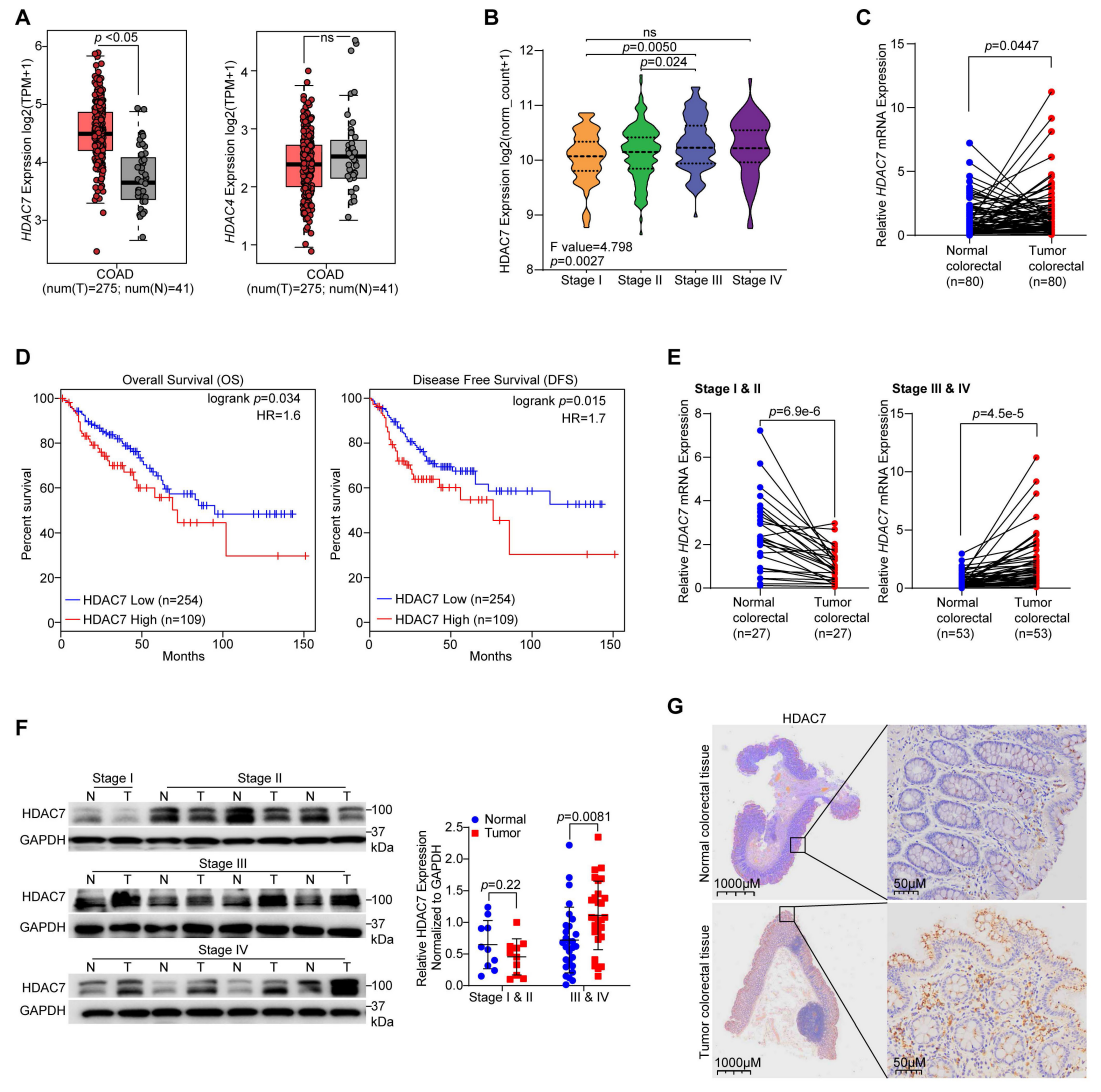
** HDAC7 is overexpressed in CRC and correlates with advanced disease and poor prognosis. (A)** Analysis of TCGA data shows that *HDAC7* mRNA expression is significantly higher in CRC tumor tissues (T) compared to adjacent normal tissues (N). In contrast, HDAC4 expression shows no significant difference (One-way ANOVA, *p*-value < 0.05 for HDAC7).** (B)** *HDAC7* expression increases with advancing pathological stage (I-IV) in the TCGA colon adenocarcinoma (COAD) cohort (One-way ANOVA). **(C)** Validation of *HDAC7* mRNA overexpression in an independent cohort of 80 paired CRC patient samples (tumor vs. adjacent normal tissues). Quantitative data are mean ± S.D. *p*-values for each indicated comparison were calculated using a two-tailed paired Wilcoxon test. **(D)** Kaplan-Meier survival analysis (GEPIA database) of CRC patients stratified by *HDAC7* expression (cutoff-high% ≥ 70, cutoff-low% < 70). High HDAC7 expression is associated with significantly worse overall survival (OS) and disease-free survival (DFS). *p*-values were determined by the log-rank test. **(E)** Stage-specific analysis of *HDAC7* mRNA levels in the same 80 paired CRC patient cohort, grouped by early (T1 & T2) and late (T3 & T4) tumor stage. Quantitative data are mean ± S.D. *p*-values were derived from a two-tailed paired Student's *t*-test. **(F)** Representative Western blot (left) and quantification (right) of HDAC7 protein levels in paired CRC (T) and normal (N) tissues. Data are mean ± S.D. (n = 3); *p*-values were calculated using a two-tailed unpaired Student's *t*-test. **(G)** Representative immunohistochemistry (IHC) staining (left) and quantification (right) of HDAC7 protein in paired CRC and normal tissues. Quantitative data are mean ± S.D. (n = 3). *p*-values for each indicated comparison were calculated using a two-tailed unpaired Student's *t*-test.

To investigate the involvement of class IIa HDACs in CRC, we examined their gene expression patterns using TCGA (The Cancer Genome Atlas) and GEPIA (Gene Expression Profiling Interaction Analysis) databases. We found distinct dysregulation patterns among class IIa HDAC members in colon adenocarcinoma (TCGA-COAD).* HDAC7* was uniquely overexpressed in tumor tissues compared to adjacent normal mucosa, while *HDAC9* showed moderate downregulation and *HDAC4* and *HDAC5* showed no significant change (**Figure 1A**, **S1A**). Furthermore, *HDAC7* expression was positively associated with advanced pathological stages (I-IV) in colon adenocarcinoma (COAD) (**Figure 1B**), with a more pronounced association than observed in rectal adenocarcinoma (READ) (**Figure S1A**). These patterns implicate HDAC7 as a key class IIa HDAC linked to CRC progression.

To validate our bioinformatics findings, we next analyzed HDAC7 expression in an independent cohort of 80 paired CRC patient samples (tumor vs. adjacent normal tissues) collected from the First Hospital of Jilin University. Consistent with bioinformatics data, *HDAC7* expression was markedly upregulated in CRC tumor tissues compared with matched non-cancerous tissues (**Figure 1C**). In contrast, *HDAC5* and *HDAC9* were downregulated and *HDAC4* expression remained unchanged (**Figure S1B**). Importantly, survival analysis using GEPIA revealed that high *HDAC7* expression correlated with reduced overall survival (OS) and disease-free survival (DFS) in CRC patients (**Figure 1D, S1C**), whereas no such association was observed for *HDAC4*, *HDAC5,* or *HDAC9*. These clinical data identify HDAC7 as the most prognostically relevant class IIa HDAC in CRC.

Notably, HDAC7 upregulation was stage-dependent. Both HDAC7 mRNA and protein levels were significantly elevated in patients diagnosed with CRC stages III and IV, whereas levels were moderately decreased in stages I and II relative to adjacent normal tissues (**Figure 1E, 1F**). Immunohistochemical (IHC) tests in three representative pairs of CRC tissues confirmed consistent HDAC7 protein overexpression in tumor tissues versus the matched adjacent normal tissues (**Figure 1G**). Additionally, clinicopathological correlation analysis in our 80-patient cohort demonstrated a strong association between *HDAC7* overexpression and both TNM stage (tumor, node, metastasis) (*p* = 5e-8) and lymph node metastasis (*p* = 0.0004). In contrast, *HDAC7* expression showed no significant relationship with patient age, gender, distant metastasis, tumor size, or molecular markers such as Ki67 and p53 status (**Table 1**). Collectively, these findings indicate a clear correlation between HDAC7 overexpression and poor patient survival and tumor progression. The stage-specific expression pattern suggests that HDAC7 may play distinct roles in early tumor suppression versus late-stage progression, warranting further investigation into its phase-dependent functions in CRC.

### HDAC7 promotes CRC proliferation, survival, and migration *in vitro* and *in vivo*

**Figure 2:**
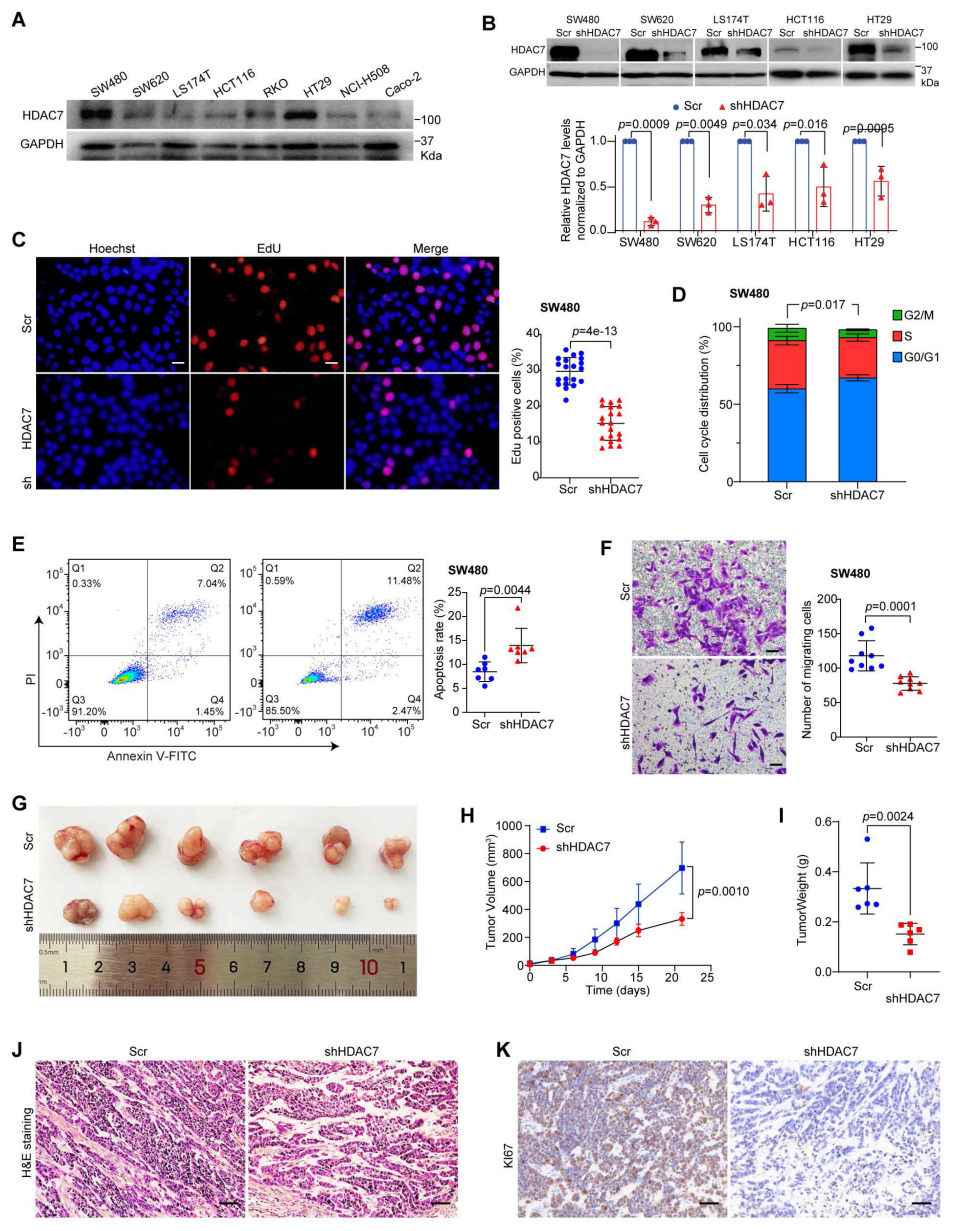
** HDAC7 promotes CRC cell proliferation, survival and migration *in vitro* and tumorigenicity *in vivo*. (A)** Western blot analysis of HDAC7 protein levels across eight CRC cell lines (normalized to GAPDH). **(B)** Validation of stable HDAC7 knockdown by shRNA (shHDAC7) in SW480, SW620, LS174T, HCT116 and HT29 cells compared to a scramble control (Scr). Quantified protein levels are mean ± S.D. (n = 3). *p*-values for each indicated comparison were derived from two-tailed unpaired Student's *t*-test.** (C)** EdU incorporation assay in SW480 cells showing that stable HDAC7 knockdown reduces proliferation. Representative images show EdU-positive nuclei (red) and total nuclei (Hoechst, blue). Scale bar, 100 µm. Quantitative data are mean ± S.D. (n = 20 fields).* p*-values were calculated using a two-tailed unpaired Student's *t*-test. **(D)** Cell cycle analysis by Annexin V/PI staining and flow cytometry in SW480 cells. Stable shRNA-mediated HDAC7 knockdown increases the proportion of G0/G1-phase cells and decreases the S-phase population. Quantitative data are mean ± S.D. (n ≥ 3). *p*-values were determined by a two-tailed unpaired Student's *t*-test. **(E)** Apoptosis analysis by flow cytometry (Annexin V-FITC/PI staining). Stable shRNA-mediated HDAC7 knockdown increases the percentage of apoptotic SW480 cells. Quantitative data are mean ± S.D. (n = 7). *p*-values were calculated using a two-tailed unpaired Student's *t*-test. **(F)** Transwell migration assay. Stable HDAC7 knockdown impairs SW480 cell migration capability. Migrated cells were stained with crystal violet. Scale bar, 100 μm. Quantified data are mean ± S.D. (n ≥ 3). *p*-values were determined by a two-tailed unpaired Student's *t*-test. **(G)** Representative images of subcutaneous xenograft tumors formed in Balb/C nude mice by SW480 cells stably expressing Scr or shHDAC7. **(H, I)** Tumor volume curves (H) and final tumor weights (I) demonstrate that stable HDAC7 knockdown suppresses tumorigenicity *in vivo*. The tumor volumes were measured every three days (H**)** and the tumor weights (I) were analyzed after excising the tumors. Quantitative data are mean ± S.D. (n ≥ 3). *p*-values were determined by a two-tailed unpaired Student's *t*-test.** (J)** Representative H&E-stained images for xenograft tumor sections of indicated groups. Scale bar, 50 μm.** (K)** Representative immunohistochemistry (IHC) staining for the proliferation marker Ki67 in xenograft tumor sections. Scale bar, 50 μm.

To explore the functional role of HDAC7 in CRC progression, we first analyzed class IIa HDAC expression across eight CRC cell lines representing distinct molecular subtypes: SW620 (lymph node metastatic derivative), SW480 (primary colon carcinoma), LS174T, HCT-116, RKO, HT29 (CMS3 metabolic subtype), NCI-H508 and Caco-2 (CMS2 epithelial subtype) (**Figure 2A**). Consistent with our clinical findings (**Figure 1E, F**), HDAC7 expression levels were elevated in SW480 and HT29 cells compared to other cell lines (**Figure 2A**). In contrast, other class IIa HDACs showed variable expression patterns. HDAC5 was upregulated in SW480 cells compared to SW620 cells, whereas HDAC4 and HDAC9 protein levels were downregulated in SW480 compared to those in SW620 (**Figure S2A**). This establishes HDAC7 as the predominant dysregulated class IIa HDAC in CRC 35.

To explore HDAC7's functional role in CRC cells, we next performed genetic knockdown of HDAC7‌ in five CRC cell lines (SW480, SW620 and LS174T, HCT116, and HT29) using shRNA (shHDAC7) (**Figure 2B, S2B**). Stable shRNA-mediated HDAC7 depletion significantly impaired proliferation in SW480, SW620, HCT116 and LS174T cells compared to non-targeting controls (**Figure 2C, S2C, S2D**), and reduced colony-forming ability in SW480, SW620, and LS174T cells (**Figure S2E-G**). These results indicate that HDAC7 is broadly required for CRC cell growth. Flow cytometry analysis of cell cycle distribution and apoptosis showed that HDAC7 depletion induced G1 cell cycle arrest, reduced S-phase populations, and increased apoptosis rates in SW480, SW620 and LS174T cells (**Figure 2D, 2E, S2H, S2I**). Transwell migration assay further demonstrated that HDAC7 knockdown substantially impaired the migratory capacity of SW480 cells compared to controls (**Figure 2F**). To further corroborate these findings and explore HDAC7's oncogenic mechanisms, we performed two independent siRNA transient knockdowns of HDAC7 (siHDAC7#1 and siHDAC7#2) in SW480 cells. Both siRNAs similarly reduced *HDAC7* expression, and reproduced the anti-proliferative, pro-apoptotic, and anti-migratory effects (**Figure S2J-M**). Taken together, these findings demonstrate that HDAC7 promotes growth, survival, and metastatic potential in CRC cells.

To evaluate HDAC7's role in tumorigenesis *in vivo*, we further established a mouse xenograft model by subcutaneously injecting stable HDAC7-knockdown SW480 cells (shHDAC7) into immunodeficient nude mice. Xenograft tumors derived from HDAC7-knockdown SW480 cells exhibited significantly delayed growth, reduced final volume, and lower weight compared to controls at the end point (**Figure 2G-I**). Histopathological analysis of H&E-stained tumor tissues revealed that stable HDAC7-depleted tumors contained fewer distinct mitotic structures, more membrane irregularities and apoptotic cells compared to the control group (**Figure 2J**), which is consistent with our cellular apoptosis findings (**Figure 2E**). Additionally, IHC analysis confirmed reduced Ki67 staining in HDAC7-knockdown tumors (**Figure 2K**), indicating that HDAC7 loss impairs proliferative capacity and tumor growth *in vivo*. Collectively, these results demonstrate that HDAC7 plays a pro-oncogenic role in CRC tumorigenesis by promoting proliferation and suppressing apoptosis.

### HDAC7 drives CRC progression via ATF3 repression and oncogenic pathway activation

**Figure 3:**
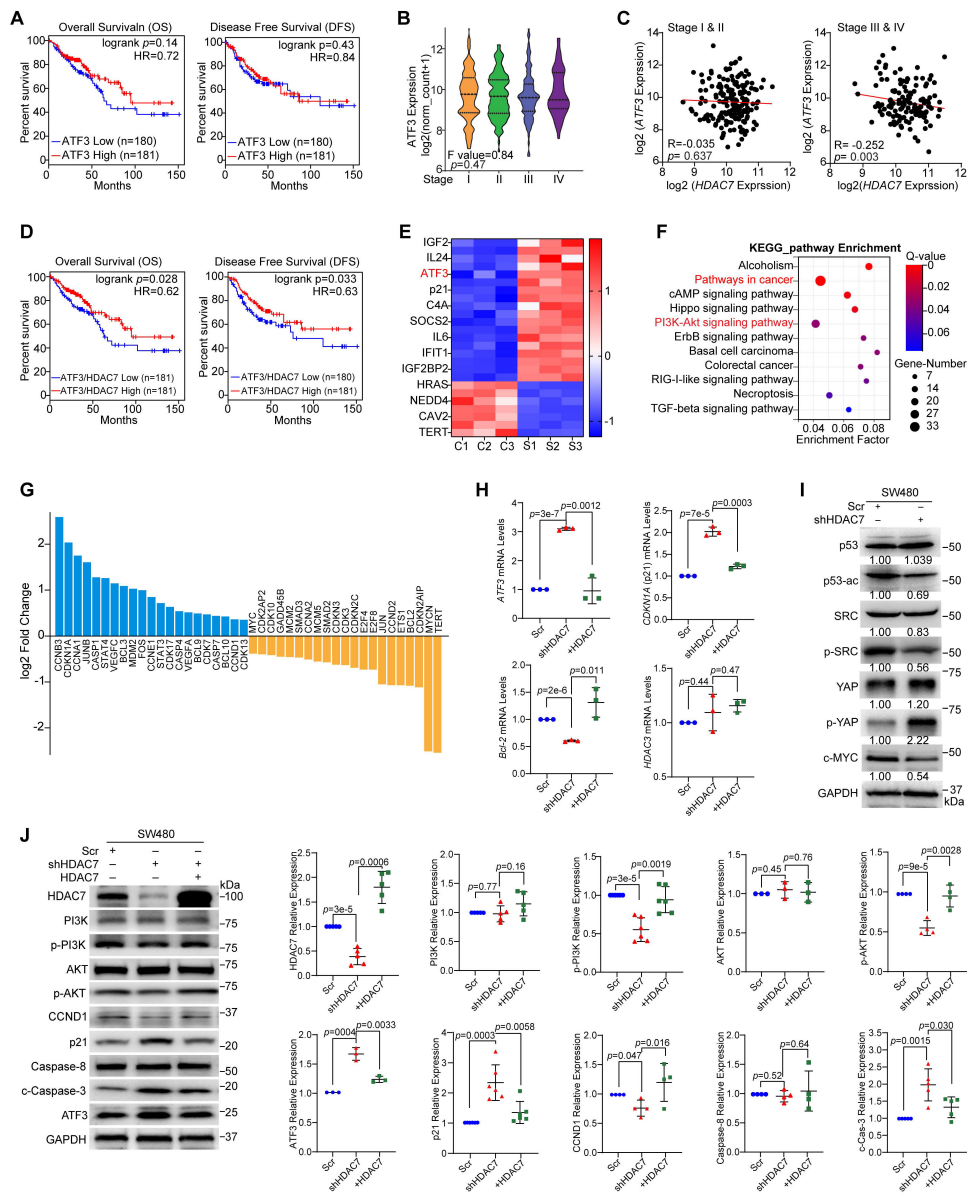
** HDAC7 represses ATF3 and activates oncogenic signaling pathways in CRC. (A)** Kaplan-Meier analysis shows that *ATF3* expression alone is not significantly associated with overall survival (OS) or disease-free survival (DFS) in CRC patients (GEPIA database). (cutoff-high% ≥ 50, cutoff-low% < 50). *p*-values are determined by the log-rank test. **(B)** *ATF3* mRNA expression decreases with advancing pathological stage (I-IV) in the TCGA CRC cohort (One-way ANOVA). **(C)** Correlation analysis between *HDAC7* and *ATF3* expression reveals a significant inverse correlation in late-stage (III & IV) CRC, but not in early-stage (I & II) disease. Pearson correlation coefficients (R) and *p*-values are indicated. **(D)** Kaplan-Meier survival analysis of CRC patients from GEPIA database stratified by *ATF3*/*HDAC7* expression ratio. (cutoff-high% ≥ 50, cutoff-low% < 50). A low *ATF3*/*HDAC7* ratio is associated with significantly worse OS and DFS (log-rank test). **(E)** Heatmap of significantly dysregulated genes in SW480 cells following stable HDAC7 knockdown (shHDAC7) compared to scramble control (Scr) (Q value ≤0.0001, |log_2_FC| ≥ 1.4).** (F)** KEGG pathway enrichment analysis of differentially expressed genes in SW480 cells upon stable HDAC7-knockdown (shHDAC7) (|log_2_FC| ≥ 1, Q value ≤0.01). Bubble size represents gene count; color indicates enrichment significance. **(G)** Histogram displaying Log_2_ fold-changes of key differentially expressed genes involved in cell cycle and apoptosis regulation upon stable shRNA-mediated HDAC7 knockdown. **(H)** qRT-PCR validation of gene expression changes in stable HDAC7 knockdown SW480 cells. Expression was normalized to *GAPDH*. Quantitative data are shown as mean ± S.D. (n ≥ 3). *p*-values were derived from a two-tailed unpaired Student's *t*-test. **(I)** Western blot analysis of key signaling proteins in control (Scr) and stable HDAC7-knockdown (shHDAC7) SW480 cells (normalized to GAPDH). **(J)** Rescue experiment showing that re-expression of HDAC7 in knockdown SW480 cells reverses the molecular changes induced by HDAC7 depletion. The relative expression was normalized by GAPDH. Data are mean ± S.D. (n ≥ 3). *p*-values were assessed by a two-tailed unpaired Student's *t*-test.

Emerging evidence indicates that ATF3 is a context-dependent transcription factor that can function as either a tumor suppressor or an oncogene in cancer 30, 36. To understand its role in CRC progression and its relationship with HDAC7, we analyzed public transcriptomic TCGA-GEPIA data. While *ATF3* expression alone did not correlate with OS and DFS in CRC cohorts (**Figure 3A**), nor differ significantly between tumor and adjacent normal tissues (**Figure S3A**), we observed a stage-dependent decrease in ATF3 levels within the COAD cohort. Specifically, lower *ATF3* expression was associated with more advanced pathological stages (Stages I-IV) (**Figure 3B**), suggesting that ATF3 deficiency may contribute to CRC progression. Notably, this stage-dependent reduction inversely paralleled the stage-associated increase in HDAC7 expression (**Figure 1E, F**), suggesting a potential regulatory interplay between these regulators.

Building on these findings, we next analyzed the transcriptome profiles from the TCGA database and observed a stage-specific inverse correlation between *HDAC7* and *ATF3* expression (*p* = 0.003) in advanced-stages (III and IV) CRC tumors but not early-stages (I and II) (**Figure 3C**). Clinically, patients with a low *ATF3*/*HDAC7* expression ratio exhibited poorer prognosis, with significantly worse OS (*p* = 0.028) and DFS (*p* = 0.033) (**Figure 3D, S3B** *vs.*** Figure 1D**). These findings suggest that this ratio is a potent prognostic marker.

To elucidate the mechanistic relationship between HDAC7 and ATF3 in transcriptional regulation, we performed RNA sequencing (RNA-seq) in HDAC7-knockdown SW480 cells (**Figure 2B**,** C**). Transcriptome profiling identified 530 upregulated and 161 downregulated genes following stable HDAC7 knockdown (fold change > 1.3, *p* < 0.05; **Figure S3C**). Importantly, *ATF3* was among the most upregulated genes following HDAC7 depletion (*p* < 0.0001; **Figure 3E**), indicating that HDAC7 is a direct transcriptional repressor of ATF3 22. To identify pathways regulated by HDAC7, we performed Kyoto Encyclopedia of Genes and Genomes (KEGG) and Gene Set Enrichment Analysis (GSEA) on differentially expressed genes (DEG) following HDAC7 knockdown. These analyses highlighted significant enrichment in oncogenic signaling, particularly the PI3K-Akt and Hippo signaling pathways (**Figure 3F, S3D, S3E**). Additionally, HDAC7 knockdown led to the coordinated downregulation of the anti-apoptotic factor *Bcl-2* and upregulation of the cell cycle inhibitor *CDKN1A* (encoding p21) (**Figure 3G**).

We validated these findings through qPCR and Western blot analysis, and confirmed that HDAC7 knockdown increased ATF3 and p21 levels and decreased* Bcl-2* mRNA and protein levels in SW480 cells (**Figure 3H, S2I**). Re-expression of wild-type HDAC7 partially reversed these effects (**Figure 3H**). At protein levels, HDAC7 depletion decreased phosphorylation of p-PI3K p85 (Tyr458), p-Akt (Ser473), p53ac, p-SRC (Tyr416) and decreased Cyclin D1 protein levels, while increasing ATF3, p21, p-YAP (Ser127) and the cleaved caspase-3 (c-Caspase-3) protein levels in SW480 cells (**Figure 3I, 3J, S3F, S3G**). These changes in p-PI3K p85 (Tyr458), p-Akt (Ser473), Cyclin D1, ATF3, p21 and c-Caspase-3 were partially rescued by HDAC7 re-expression (**Figure 3J**), confirming HDAC7 plays a direct regulatory role in these pathways. Notably, *HDAC3* mRNA levels were unaffected by modulating HDAC7 (**Figure 3H**), indicating that HDAC7 regulates these pathways without altering HDAC3 levels.

### HDAC7 represses ATF3 to promote oncogenic signaling in CRC cells

**Figure 4:**
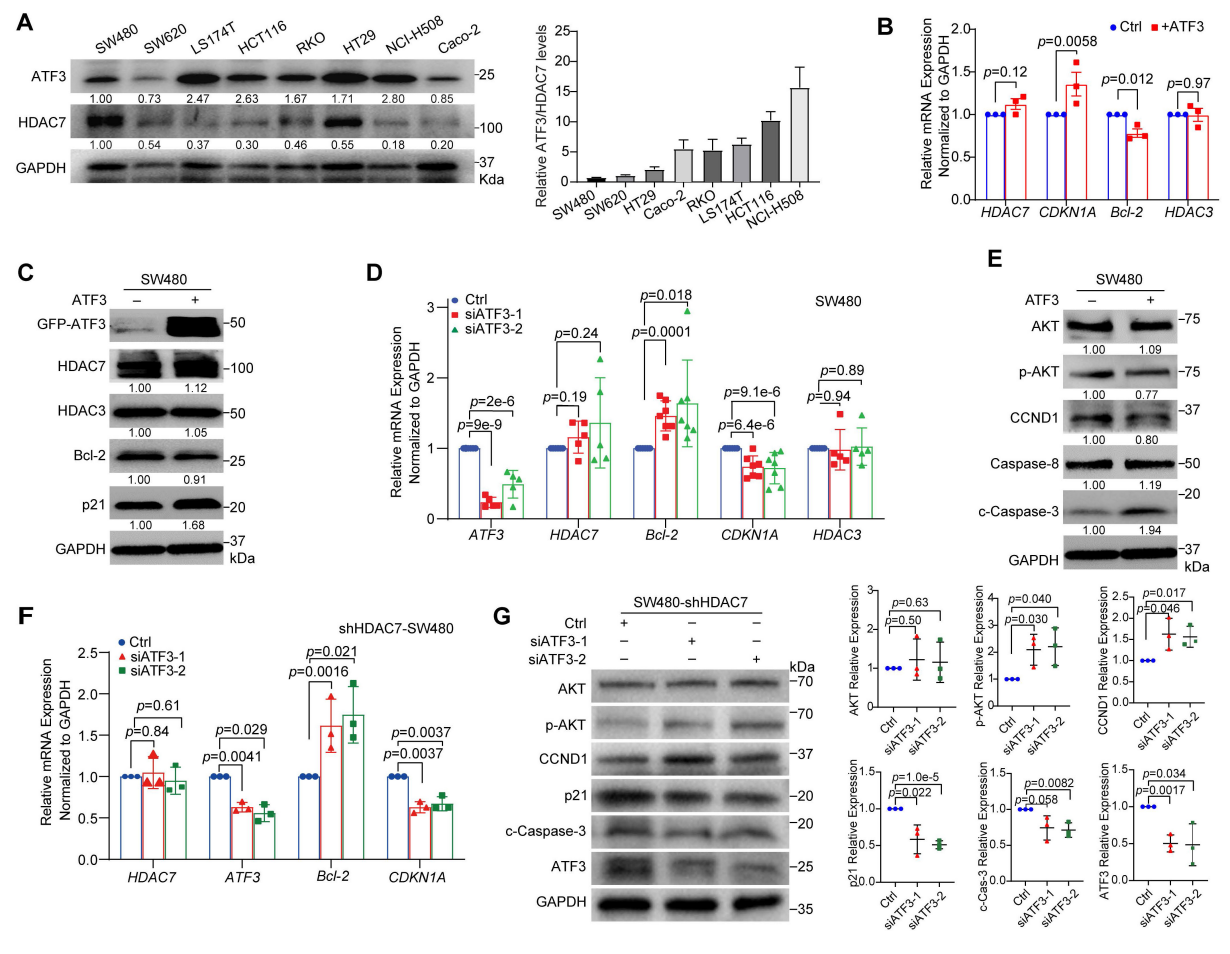
** HDAC7 represses ATF3 to promote oncogenic signaling in CRC cells. (A)** Western blot analysis of endogenous ATF3 and HDAC7 protein levels across eight CRC cell lines (left). Quantification (mean values, n ≥ 3) of the ATF3/HDAC7 protein ratio (right), showing a pronounced reduction in invasive SW480 and SW620 cells. The HDAC7 and GAPDH loading controls are the same as those shown in Figure 2A, as both analyses were performed on the same samples. **(B, C)** ATF3 overexpression in SW480 cells increases *CDKN1A* (p21) and decreases *Bcl-2* mRNA (B) and protein (C) levels, without affecting HDAC7 or HDAC3. Data are mean ± SD (n ≥ 3). *p*-values were calculated using a two-tailed unpaired Student's *t*-test. **(D)** siRNA-mediated transient ATF3 knockdown (siATF3-1, siATF3-2) in SW480 cells decreases *CDKN1A* and increases *Bcl-2* mRNA, confirming its regulatory role, while *HDAC7* and *HDAC3* levels remain unchanged. Quantitative data are mean ± S.D. (n = 5; two-tailed unpaired Student's *t*-test).** (E)** Western blot analysis shows ATF3 overexpression suppresses PI3K-Akt signaling (reduced p-Akt and Cyclin D1) and promotes apoptosis (increased Caspase-8 and cleaved Caspase-3). Data are mean ± S.D. (n ≥ 3; two-tailed unpaired *t*-test). **(F, G)** Genetic relationship analysis. siRNA-mediated transient co-depletion of ATF3 in HDAC7-knockdown SW480 cells reverses the tumor-suppressive molecular phenotype, partially restoring *Bcl-2* and reducing *CDKN1A* mRNA (F) (Data are mean ± S.D.; n ≥ 3; two-tailed unpaired *t*-test), while reactivating PI3K-Akt signaling (increased p-Akt and Cyclin D1) and diminishing apoptosis (decreased cleaved caspase-3) at the protein level (G). This establishes ATF3 as the essential downstream effector.

To explore the mechanism of HDAC7-mediated regulation of ATF3 in CRC cells, we first analyzed ATF3 protein levels across eight CRC cell lines (**Figure 4A**). ATF3 expression levels were notably lower in the more invasive SW620 and SW480 cells than in Caco-2, LS174T, HCT-116, RKO, HT29 and NCI-H508 cells (**Figure 4A**). Importantly, the ATF3/HDAC7 ratio was markedly reduced in these SW480 and SW620 cells compared to other cell lines (**Figure 4A**). This cell-based quantification is consistent with our clinical observation that a decreased ATF3/HDAC7 ratio correlates with poor patient outcomes (**Figure 3D**), and suggests that HDAC7-mediated ATF3 repression is linked to aggressive disease.

We next investigated ATF3's functional role. Overexpression of GFP-tagged ATF3 (GFP-ATF3) in both SW480 and LS174T cells upregulated *CDKN1A* (p21) mRNA and protein levels and downregulated Bcl-2 expression (**Figure 4B, 4C, S4A, S4B**). Conversely, siRNA-mediated ATF3 knockdown (siATF3-1, siATF3-2) produced the opposite effect (**Figure 4D, S4C**). Additionally, Western blot analysis showed that GFP-ATF3 overexpression also suppressed oncogenic PI3K-Akt signaling (reduced p-Akt and Cyclin D1) and promoted apoptosis (increased cleaved Caspase-3) (**Figure 4E, S4A**). Importantly, ATF3 overexpression or knockdown did not alter HDAC7 or HDAC3 expression levels (**Figure 4B-D, S4A-C**), positioning HDAC7 and HDAC3 as upstream regulators. These findings underscore ATF3's critical role in regulating *Bcl-2* and *CDKN1A* transcription, thereby influencing key survival and cell cycle pathways in CRC cells 23, 34.

This upstream relationship was confirmed through a genetic rescue experiment. siRNA-mediated co-depletion of ATF3 in HDAC7-knockdown cells reversed the tumor-suppressive effects induced by HDAC7 depletion. Specifically, it partially restored *Bcl-2* expression and reduced *CDKN1A* levels (**Figure 4F**). Furthermore, Western blot analysis revealed that ATF3 co-silencing reactivated PI3K-Akt signaling (increased p-Akt and Cyclin D1), and diminished apoptosis (decreased cleaved caspase-3) (**Figure 4G**). This establishes ATF3 as the essential downstream effector mediating the anti-tumor impact of HDAC7 loss.

### HDAC7 scaffolds a catalytic-independent repressive complex at the *ATF3* locus

**Figure 5:**
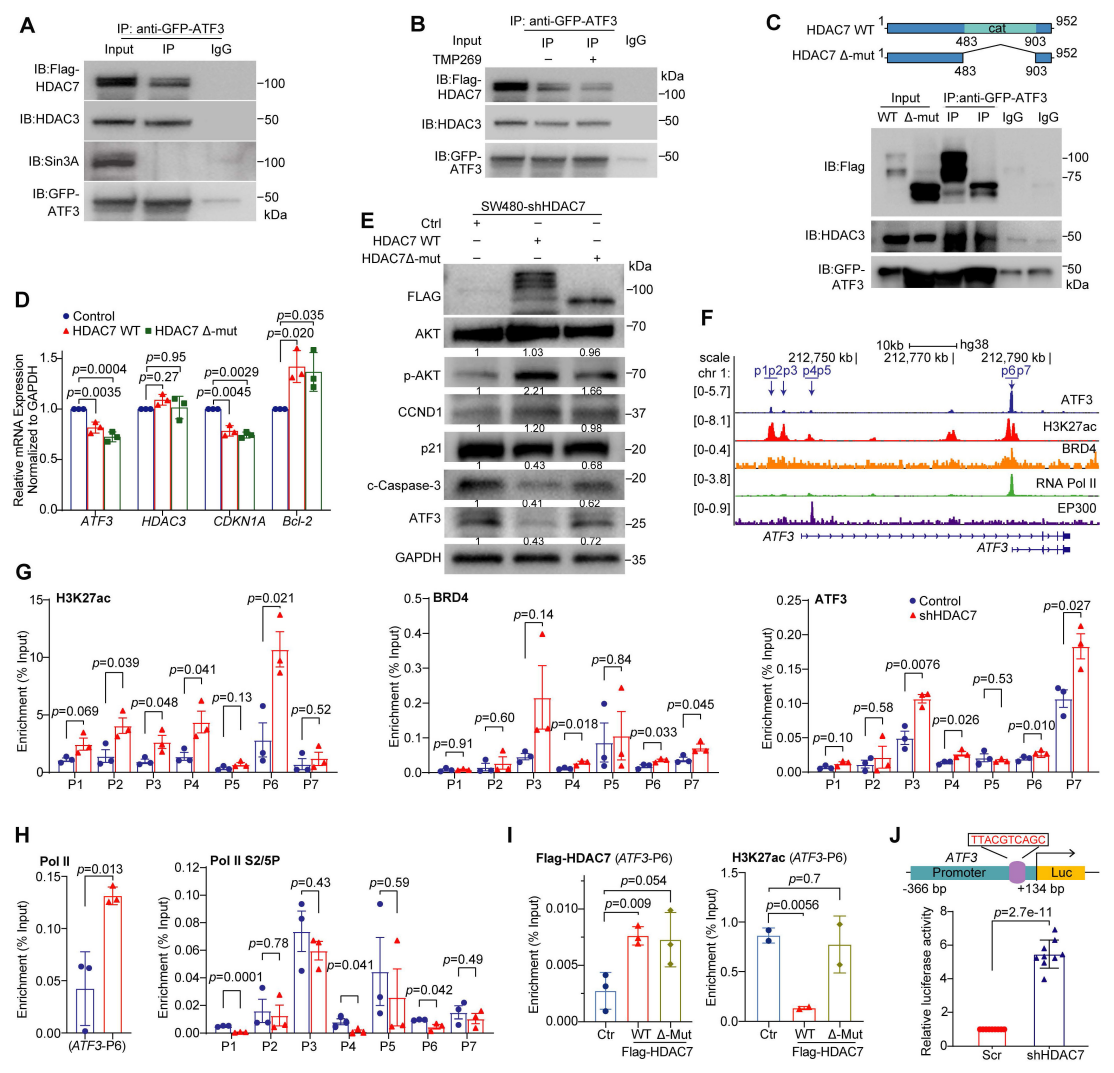
** HDAC7 scaffolds a repressive complex at the ATF3 locus to control its transcriptional output. (A-C)** Co-immunoprecipitation (Co-IP) assays demonstrate the physical interaction between HDAC7, HDAC3, and ATF3. (A) GFP-ATF3 co-precipitates with Flag-HDAC7 and endogenous HDAC3 in HEK293T cells. (B) The Class IIa HDAC inhibitor TMP269 does not disrupt the HDAC7-ATF3-HDAC3 interaction. (C) A catalytic domain-deletion mutant of HDAC7 (Δ-Mut) retains binding to ATF3 and HDAC3, confirming a scaffolding mechanism independent of deacetylase activity. **(D, E)** Functional rescue of HDAC7 depletion. Re-expression of wild-type (WT) HDAC7, but not the Δ-Mut, in HDAC7-knockdown SW480 cells restores the repressive molecular phenotype, as shown by qRT-PCR (D) (n = 3; two-tailed unpaired Student's *t*-test) and Western blot (E). This indicates the catalytic domain contributes to optimal repressor function. Data are mean ± S.D. **(F)** Public ChIP-seq tracks (GEO datasets) show co-occupancy of ATF3 with active chromatin marks (H3K27ac) and transcriptional co-activators (BRD4, Pol II, p300) at enhancer (p1-p3) and promoter (p4-p7) regions of the *ATF3* locus. **(G, H)** ChIP-qPCR analysis in SW480 cells. Stable HDAC7 knockdown does not alter ATF3 occupancy but significantly increases enrichment of active marks (H3K27ac, H3K18ac) and co-activators (BRD4, Pol II) at ATF3 regulatory regions (G, H). It also decreases phosphorylation of Pol II (S2P/S5P) (H), indicating a shift in transcriptional state. Data are mean ± S.E.M. (n = 3; two-tailed unpaired *t*-test). **(I)** ChIP-qPCR shows that re-expression of WT HDAC7, but not the Δ-Mut, reverses the HDAC7 knockdown-induced increase in H3K27ac, despite similar chromatin occupancy. This confirms the catalytic domain's role in recruiting co-repressor activity. Data are mean ± S.E.M. (n ≥ 2; two-tailed unpaired *t*-test).** (J)** ATF3 promoter-driven luciferase reporter assay. Schematic depiction of the ATF3 promoter-driven luciferase reporter construct (top). HDAC7 knockdown significantly increases reporter activity in SW480 cells (bottom), confirming enhanced ATF3 transcription. The pGL3 plasmid construct carrying the ATF3 core promoter region (-366 - +134 bp) was transfected into Scr or shHDAC7 knockdown SW480 cells. Luciferase activity was normalized to *Renilla* luciferase activity. Quantitative data (bottom) are shown as mean ± S.D. (n ≥ 9). *p*-values are calculated using a two-tailed unpaired Student's *t*-test.

To understand the molecular mechanism underlying HDAC7-mediated repression, we first investigated complex formation. Co-immunoprecipitation (Co-IP) assays showed that GFP-tagged ATF3 was associated with HDAC7 and the class I deacetylase HDAC3, but not the co-repressor SIN3A, in HEK293T cells (**Figure 5A**, **S4D**). Endogenous Co-IP confirmed HDAC7 presence in ATF3 immunocomplexes in SW480 cells (**Figure S4E**). Notably, HDAC7 knockdown attenuated the ATF3-HDAC3 interaction (**Figure S4F**), indicating that HDAC7 acts as a scaffold to stabilize the ternary HDAC7-HDAC3-ATF3 complex.

To investigate whether this scaffolding interaction depends on HDAC7's deacetylase activity, we next used the class IIa HDAC inhibitor TMP269 and the HDAC7 catalytic domain deletion mutant (Δ-Mut). Neither pharmacological inhibition (TMP269) nor expression of the catalytically dead mutant (Δ-Mut) disrupted the ATF3-HDAC7/HDAC3 complex formation (**Figure 5B, 5C**), suggesting that the complex assembly occurs independently of catalysis. However, functional rescue experiments revealed a role for the catalytic domain in repressive activity. Re-expression of wild-type (WT) HDAC7 in knockdown cells restored the repressive phenotype, reversing the ATF3 and *CDKN1A* (p21) upregulation and Bcl-2 downregulation. In contrast, the Δ-Mut showed markedly weaker rescue capacity (**Figure 5D, 5E**). These results indicate that while dispensable for complex assembly, the catalytic domain contributes to repressor function, likely by stabilizing the complex.

We then validated the functional importance of HDAC3 within this axis. siRNA-mediated HDAC3 knockdown (siHDAC3-1, siHDAC3-2) phenocopied HDAC7 loss, increasing ATF3 and p21 (*CDKN1A*) expression while decreasing* HDAC7* and* Bcl-2*, and suppressing oncogenic PI3K-Akt signaling (**Figure S5A**, **S5B**). Functional assays validated that HDAC3 depletion also reduced colony formation (**Figure S5C**) and promoted apoptosis (**Figure S5D**). Notably, combined knockdown of HDAC7 and HDAC3 produced an additive tumor-suppressive effect (**Figure S5E, S5F**), underscoring their cooperative role in sustaining the repressive state.

To understand the transcriptional consequences of this complex at chromatin, we integrated public ChIP-seq datasets from the Gene Expression Omnibus (GEO). Bioinformatics analysis revealed that ATF3-binding sites (GSE32465) colocalize with regions enriched for active chromatin marks (H3K27ac; GSE143653) and co-activators such as BRD4 (GSE73319), p300 (GSE51176), and Pol II (GSE179540) (**Figure 5F**) 37-41. This suggests an intrinsic potential for ATF3 autoregulation. Consistent with this, ChIP-qPCR in SW480 cells showed that HDAC7 knockdown generally did not displace ATF3 from its own promoter. Instead, it substantially increased the recruitment of BRD4 and Pol II and the enrichment of H3K27ac and H3K18ac at *ATF3* regulatory loci (**Figure 5G, 5H, S4G**). HDAC7 depletion also reduced levels of phosphorylated Pol II at serine-2 and -5 (S2P and S5P), reflecting a shift in transcriptional state 42.

To directly examine HDAC7 occupancy, we performed ChIP using Flag-tagged constructs. Both WT HDAC7 and the Δ-Mut showed comparable enrichment at the *ATF3* promoter (**Figure 5I**), confirming that chromatin binding is mediated by the N-terminal domain independently of catalytic function. Despite equivalent promoter occupancy, only re-expression of WT HDAC7, but not the Δ-Mut, reversed the HDAC7 knockdown-induced accumulation of H3K27ac at the locus (**Figure 5I**). This demonstrates that the catalytic domain is required for co-repressor activity and histone deacetylation. Furthermore, we constructed an *ATF3* promoter-driven luciferase reporter assay in SW480 cells, in which HDAC7 depletion enhanced the reporter activity, consistent with derepressed ATF3 transcription (**Figure 5J**).

In summary, ATF3's transcriptional output is determined by a competitive balance between co-activator and co-repressor complexes. In CRC, elevated HDAC7 scaffolds a repressive complex with HDAC3 and ATF3 at the *ATF3* locus, limiting co-activator recruitment and histone acetylation to maintain transcriptional silencing. Depletion or inhibition of HDAC7 disrupts the scaffold and shifts this equilibrium, enabling ATF3 to recruit co-activators BRD4 and Pol II, elevate H3K27ac, and initiate a self-activating loop that promotes a tumor-suppressive program.

### Pharmacological inhibition of HDAC7 promotes ATF3 autoregulation by facilitating BRD4 and Pol II recruitment

**Figure 6:**
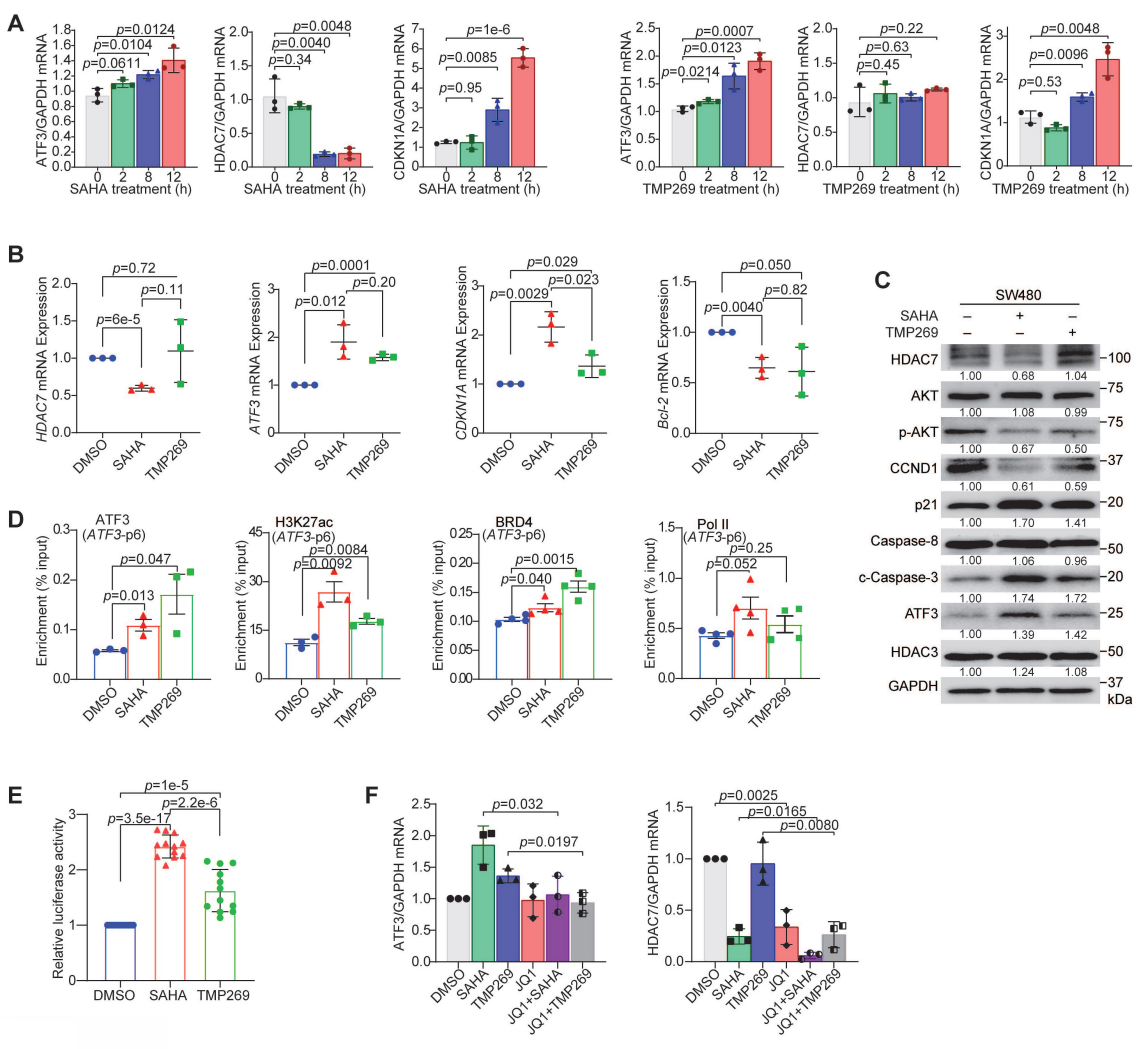
** Pharmacological inhibition of HDAC7 reactivates ATF3-mediated tumor suppression. (A)** Time-course qRT-PCR analysis of *ATF3*, *HDAC7* and* CDKN1A* (p21) mRNA levels in SW480 cells treated with SAHA (1.5 µM, left) and TMP269 (3 µM, right). Data are mean ± S.D. (n = 3; two-tailed unpaired Student's *t*-test).** (B)** qRT-PCR analysis of *HDAC7*, *CDKN1A*, *Bcl-2* and *ATF3* mRNA levels after 24-h treatment with SAHA (1.5 µM) or TMP269 (3 µM) in SW480 cells. Data are mean ± S.D. (n ≥ 3; two-tailed unpaired Student's *t*-test). **(C)** Western blot analysis of indicated proteins after SAHA (1.5 µM) or TMP269 (3 µM) treatment in SW480 cells (normalized to GAPDH). Data are shown as mean of three independent experiments. **(D)** ChIP-PCR analysis of H3K27ac, ATF3, BRD4, and Pol II enrichment at *ATF3* regulatory regions in SW480 cells following treatment with DMSO vehicle (0.05%), SAHA (1.5 µM) or TMP269 (3 µM). Data are mean ± S.E.M (n ≥ 3; two-tailed unpaired Student's *t*-test).** (E)** *ATF3* promoter-driven luciferase activity after treatment with SAHA (1.5 µM) or TMP269 (3 µM) in SW480 cells (normalized to *Renilla* luciferase). Data represent mean ± S.D. (n ≥ 3; two-tailed unpaired Student's *t*-test).** (F)** qRT-PCR analysis of *ATF3* and *HDAC7* expression in SW480 cells following treatment with SAHA (12 h), TMP269 (12 h), JQ1 (6 h) or the combination of JQ1 with SAHA or TMP269 (total 12 h). Data represent mean ± S.D. (n ≥ 3; two-tailed unpaired Student's *t*-test).

To characterize the functional consequence of HDAC7 inhibition on ATF3 regulation, we treated SW480 cells with the pan-class I/II HDAC inhibitor SAHA or the class IIa-selective inhibitor TMP269. Both inhibitors induced a time-dependent upregulation of ATF3 and its target *CDKN1A* (p21) mRNA (**Figure 6A**). While SAHA also reduced HDAC7 mRNA and protein levels, TMP269 showed no significant effects (**Figure 6A**-**C, S6A**) 43, 44, confirming that class IIa HDAC inhibition directly targets HDAC7 activity rather than its expression.

ChIP analysis revealed the underlying molecular mechanism. Both SAHA and TMP269 treatments increased chromatin enrichment of H3K27ac and H3K18ac and recruitment of ATF3, BRD4, and Pol II at the *ATF3* loci (p6, p3) (**Figure 6D, Figure S6B**). Consistent with this open chromatin state, an *ATF3* promoter-driven luciferase reporter showed enhanced activity upon inhibitor treatment (**Figure 6E**). These findings demonstrate that HDAC7 inhibition shifts the epigenetic landscape at the *ATF3* gene, promoting co-activator recruitment and transcriptional activation. Notably, SAHA generally induced a stronger upregulation of *ATF3* and *CDKN1A* (p21) (**Figure 6B**), and a greater increase in H3K27ac and Pol II enrichment, compared to TMP269 (**Figure 6D**). This differential efficacy suggests class I HDACs (notably HDAC3, a known component of the repressive complex) cooperate with class IIa HDAC7 to fully suppress ATF3 and subsequently regulate downstream targets (e.g., *CDKN1A*). Additionally, treatment with the BET bromodomain inhibitor JQ1 suppressed both ATF3 and *HDAC7* expression and completely abrogated their induction by SAHA or TMP269 (**Figure 6F**). This confirms that BRD4 is an essential, downstream effector required for the transcriptional reactivation of *ATF3* following HDAC7 inhibition.

### HDAC7 inhibition activates ATF3-mediated tumor suppression in CRC

**Figure 7:**
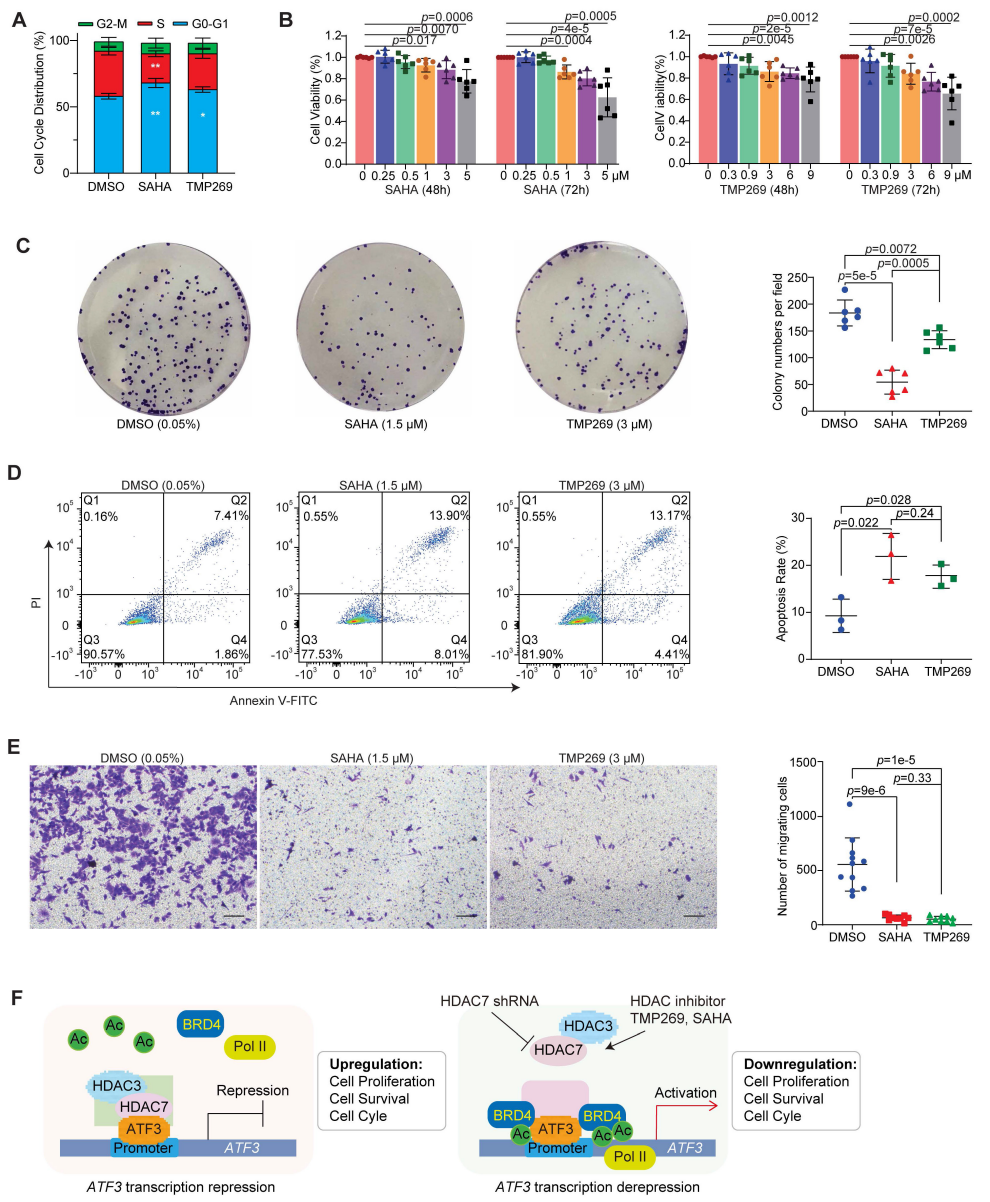
**Pharmacological inhibition of HDAC7 suppresses CRC tumorigenesis. (A)** Cell cycle distribution of SW480 cells after treatment with vehicle (0.05% DMSO), SAHA (1.5 µM) or TMP269 (3 µM). Bar charts show the percentages of cells in each phase. Quantitative data are mean ± S.D. (n ≥ 3; two-tailed unpaired Student's *t*-test; *, *p* < 0.05, **, *p* < 0.01).** (B)** Dose- and time-dependent suppression of SW480 cell viability by SAHA (*left*) and TMP269 (*right*) as measured by MTT assay. Cells were treated with indicated concentrations of SAHA (0, 0.25, 0.5, 1, 3 and 5 µM) or TMP269 (0, 0.3, 0.9, 3, 6 and 9 µM) for 48 or 72 h. Data represent mean ± SD (n ≥ 3; two-tailed unpaired Student's *t*-test).** (C)** Colony-formation assay of SW480 cells treated with DMSO (0.05%), SAHA (1.5 µM) or TMP269 (3 µM). Quantitative data are mean ± S.D. (n ≥ 3; two-tailed unpaired Student's *t*-test). **(D)** Apoptosis assessed by Annexin V/PI staining and flow cytometry in SW480 cells following treatment with 0.05% DMSO, 1.5 µM SAHA, or 3 µM TMP269. Quantitative data are shown as mean ± S.D. (n ≥ 3; two-tailed unpaired Student's *t*-test).** (E)** Transwell migration assay. SW480 cells were treated with 1.5 µM SAHA or 3 µM TMP269, and migrated cells were stained with crystal violet. Representative images (left) and quantification (right) are shown. Data are mean ± SD (n ≥ 3; two-tailed unpaired Student's *t*-test). **(F)** Mechanistic model of HDAC7-mediated epigenetic regulation of ATF3 in CRC. *Left*: In the oncogenic state, HDAC7 scaffolds a repressive complex with HDAC3 and ATF3 at the *ATF3* promoter, reducing H3K27ac and excluding Pol II and co-activators like BRD4, thereby silencing ATF3 transcription. *Right*: Genetic depletion or pharmacological inhibition of HDAC7 disrupts the complex. This enables the recruitment of BRD4 and Pol II, increases H3K27ac, and initiates a self-activating loop of ATF3 self-activation, restoring its tumor-suppressive function. ATF3 chromatin occupancy remains unchanged.

To assess the tumor-suppressive consequences of HDAC7 inhibition, we examined its effects on key signaling pathways and cellular phenotypes. In SW480 cells, treatment with SAHA or TMP269 markedly suppressed oncogenic PI3K-Akt signaling (decreased p-Akt and Cyclin D1) and activated pro-apoptotic and cell cycle inhibitory programs (increased ATF3, p21 and cleaved Caspase-3) (**Figure 6C, S6A**). Functionally, this molecular inhibition translated into potent anti-tumor effects. Both compounds induced cell cycle arrest (**Figure 7A, S7A**), suppressed proliferation across HCT-116, HT29, SW480 and SW620 cell lines (**Figure 7B, S7B, S7C, S7F**), and inhibited long-term clonogenic survival in SW480 and SW620 cells (**Figure 7C, S7D**). Furthermore, these treatments promoted apoptosis (**Figure 7D, S7E**) and impaired metastatic potential in SW480 cells (Transwell migration assay; **Figure 7E**). These coordinated effects demonstrate class IIa HDAC (e.g., HDAC7) inhibition impedes CRC progression through a multipronged mechanism: reactivating ATF3 to suppress proliferative signaling (PI3K/Akt-Cyclin D1), enforce cell cycle arrest (via p21), and trigger apoptosis (caspase-3). The efficacy of TMP269 highlights its potential as a targeted therapeutic strategy to reactivate ATF3-mediated tumor suppression in CRC.

## Discussion

CRC remains a leading cause of cancer-related mortality worldwide. Despite therapeutic advances, its rising incidence and mortality underscore the urgent need for innovative treatment strategies 45. In this study, we identify HDAC7 as a pivotal epigenetic regulator of CRC pathogenesis. We demonstrate that HDAC7 is overexpressed in CRC tumors, where its levels correlate with advanced disease stage, metastasis, and poor patient survival. Mechanistically, HDAC7 does not rely on its weak deacetylase activity but instead functions as a scaffold for a repressive complex with HDAC3 and ATF3 at *ATF3* regulatory regions. This complex reduces activating histone marks (H3K27ac/H3K18ac), excludes co-activators such as BRD4 and Pol II, and epigenetically silences *ATF3* transcription. Consequently, ATF3's tumor-suppressive functions are disabled, while augmenting oncogenic PI3K-Akt signaling (p-Akt: Ser473, Cyclin D1) and suppressing the Hippo pathway (p-YAP: Ser127).

Genetic depletion or pharmacological inhibition of HDAC7 disrupts this repressive complex, triggering a functional shift in ATF3. Once dissociated from repression, ATF3 recruits BRD4 and Pol II, increases H3K27ac at its own promoter, and initiates a self-activating loop that robustly activates its own transcription. Reactivated ATF3 then executes a tumor-suppressive pathway through downregulating Bcl-2, upregulating p21/*CDKN1A*, inducing caspase-3-mediated apoptosis, and suppressing tumor growth *in vivo*. These findings illustrate HDAC7 inhibition as a promising therapeutic strategy to reactivate ATF3-mediated tumor suppression in CRC, particularly in patients with a low ATF3/HDAC7 expression ratio, which is a potential biomarker of aggressive disease.

Our work also resolves the longstanding paradox of ATF3's context-dependent roles in cancer. We show that ATF3's function as a transcriptional repressor or activator is not intrinsic but is determined by the competitive recruitment of opposing cofactor complexes. In CRC, high HDAC7 levels scaffold a repressive HDAC3 complex, confining ATF3 in an inactive, oncogenic state (**Figure 7F**). Inhibition of HDAC7 shifts the equilibrium, allowing ATF3 to engage BRD4/Pol II/p300 co-activator complexes and transition to a tumor-suppressive, self-activating state (**Figure 7F**).

These findings offer fundamental insight into transcription factor plasticity, demonstrating how a single factor's function is governed by the local chromatin environment and cofactor availability 46, 47. This model extends beyond CRC and may represent a generalizable mechanism for other bZIP transcription factors, whereby a scaffolding protein regulates transcription factor function by dictating cofactor recruitment.

Therapeutically, our work highlights the promise of selective HDAC7 inhibitors, such as TMP269 derivatives, to stimulate ATF3 to its tumor-suppressive state. Future efforts should focus on identifying the precise cis-regulatory motifs and dimerization partners that govern ATF3's transition between repressor and activator complexes, characterizing the tissue-specific dynamics of the HDAC7-ATF3 axis in other malignancies, and ultimately validating the ATF3/HDAC7 ratio as a clinical biomarker for patient stratification.

## Supplementary Material

Supplementary figures and tables.

## Figures and Tables

**Table 1:** The relationship between clinicopathological characteristics and HDAC7 expression in CRC patients

Clinicopathologic characteristics	Number	Percent	HDAC7		Chi-square test
			High	Low	*p-*value
**Age (years)**			47	33	0.8208
< 60	33	41.25%	20	13	
≥ 60	47	58.75%	27	20	
**Gender**					0.2491
Male	48	60.00%	32	16	
Female	32	40.00%	17	15	
**TNM stage**					5e-8
I	23	28.75%	2	21	
II	38	47.50%	29	9	
III	19	23.75%	16	3	
**Lymph node metastasis**					0.0004
N0	44	55.00%	17	27	
N1	29	36.25%	22	7	
N2	7	8.75%	7	0	
**Distant metastasis**					0.4756
M0	73	91.25%	42	31	
M1	7	8.75%	5	2	
**Tumor size (cm^3^)**					0.5753
10	31	38.75%	16	15	
10-30	29	36.25%	18	11	
> 30	20	25.00%	13	7	
**Marker**					
** Ki67**					0.7293
≤ 50%	9	11.25%	6	3	
> 50%	71	88.75%	41	30	
**p53**					> 0.9999
≤ 50%	34	42.50%	20	14	
> 50%	46	57.50%	27	19	

## Data Availability

The data that support this study are available from the corresponding authors upon reasonable request. All protocols used in this study are available in the Methods sections. All RNA-seq data generated in this study are deposited in the NCBI SRA database under BioProject accession code (PRJNA1170925). Source data are provided with this paper. Supplementary Information is available in the online version of the paper.

## Abbreviations

HDACs: histone deacetylases
CRC: colorectal cancer
Co-IP: co-immunoprecipitation
HATs: histone acetyltransferases
HDACi: HDAC inhibitors
PEI: polyethyleneimine linear
ORF: open reading frame
H&E: hematoxylin and eosin
EdU: 5-ethynyl-2'-deoxyuridine
MTT: 3-[4,5-dimethylthiazol-2-yl]-2,5-diphenyl tetrazolium bromide
PI: propidium iodide
RT-qPCR: reverse transcription quantitative polymerase chain reaction
CCND1: Cyclin D1
Co-IP: co-immunoprecipitation assay
COAD: colon adenocarcinoma
READ: rectum adenocarcinoma
OS: overall survival
DFS: disease-free survival
