# Supplementary material for: HDAC7-Induced Epigenetic Repression Modulates ATF3 Functional Plasticity in Colorectal Cancer Pathogenesis
Source: Int J Biol Sci. 2026 May 1;22(9):4956–75. doi: 10.7150/ijbs.121348 (PMC13182534; doi:10.7150/ijbs.121348)
Supplement: Supplementary file 1 — Supplementary figures and tables. [file ijbsv22p4956s1.pdf]

Supplementary Table 1, Sequences of q-PCR primers

Primer Name	Primer Sequence
HDAC7-qPCR-F	F: 5’-TCCTGGCACAGCGGATGTTTGT-3’
HDAC7-qPCR-R	R: 5’-TGAAGGCGAGGTCAGTGACACT-3’
ATF3-qPCR-F	F: 5’-CGCTGGAATCAGTCACTGTCAG-3’
ATF3-qPCR-R	R: 5’-CTTGTTTCGGCACTTTGCAGCTG-3’
HDAC3-qPCR-F	F: 5’-GAGTTCTGCTCGCGTTACACAG-3’
HDAC3-qPCR-R	R: 5’-CGTTGACATAGCAGAAGCCAGAG-3’
BCL-2-qPCR-F	F: 5’-ATCGCCCTGTGGATGACTGAGT-3’
BCL-2-qPCR-R	R: 5’-GCCAGGAGAAATCAAACAGAGGC-3’
CDKN1A-qPCR-F	F: 5’-AGGTGGACCTGGAGACTCTCAG-3’
CDKN1A-qPCR-R	R: 5’-TCCTCTTGGAGAAGATCAGCCG-3’
GAPDH-qPCR-F	F: 5’-GTCTCCTCTGACTTCAACAGCG-3’
GAPDH-qPCR-R	R: 5’-ACCACCCTGTTGCTGTAGCCAA-3’

Supplementary Table 2, *ATF3* ChIP-qPCR primer sequences

Primer Name	Primer Sequence
ATF3-ChIP-P1-F	F: 5’-CTGCTGACTCATCTGACATTA-3’
ATF3-ChIP-P1-R	R: 5’-CCAGGCCTGGTCCCTTTAAGGC-3’
ATF3-ChIP-P2-F	F: 5’-GAAAACGGAAGAACAACTGAG-3’
ATF3-ChIP-P2-R	R: 5’-AGTCAAGGGCCATAGAAAACT-3’
ATF3-ChIP-P3-F	F: 5’-AGCCCCGGCAAGCAAAAGAAC-3’
ATF3-ChIP-P3-R	R: 5’-CCAGGCTATGTGTGTCTCGCTG -3’
ATF3-ChIP-P4-F	F: 5’-ATGGATCACAACTGAACAGTGA-3’
ATF3-ChIP-P4-R	R: 5’-AAGTAAAATCGCTACCCCTAG-3’
ATF3-ChIP-P5-F	F: 5’- AGCTGCCGTAGGAGCAGCATG -3’
ATF3-ChIP-P5-R	R: 5’- GCCCACTGCCGTGTAAACACT -3’
ATF3-ChIP-P6-F	F: 5’-TTGTGTTAACCGGCGGGCTTC-3’
ATF3-ChIP-P6-R	R: 5’-AGCTTCAGGAAAATACCCCCT-3’
ATF3-ChIP-P7-F	F: 5’-CCCGGGAAGCTATTAATAGCA-3’
ATF3-ChIP-P7-R	R: 5’-AGAAGAGAGCTGTGCAGTGC-3’

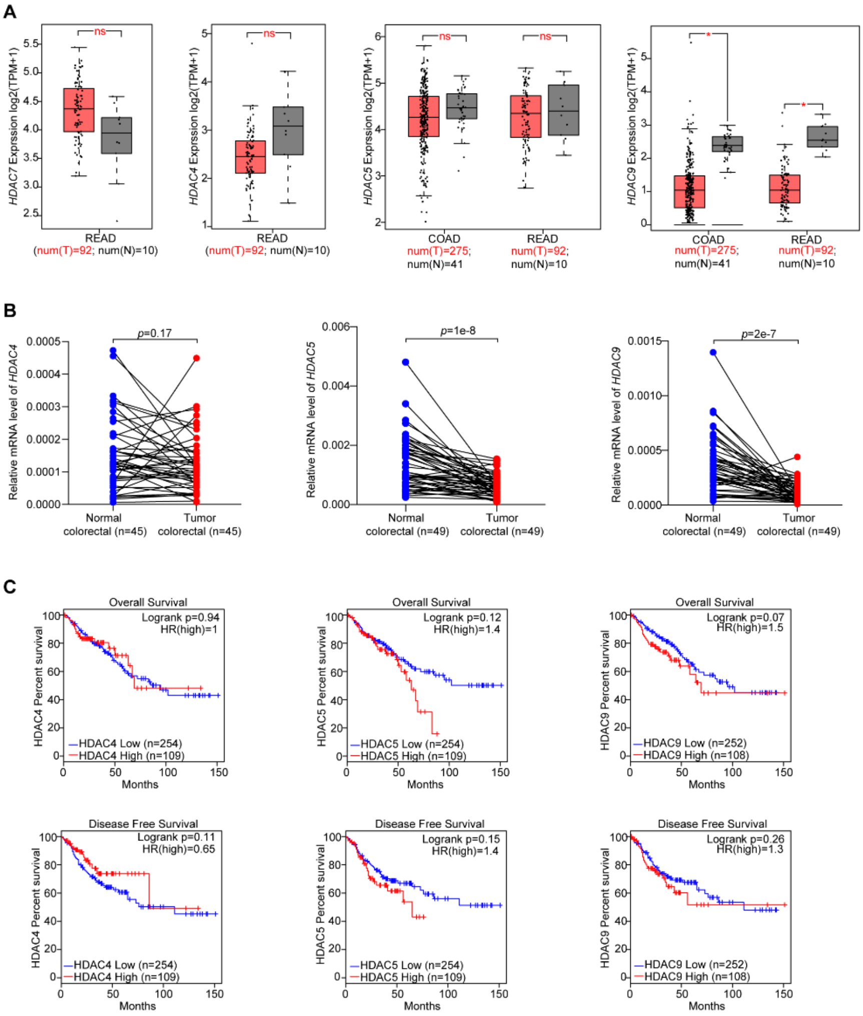

Figure S1. Class IIa HDACs expresstion and survival curves in CRC from TCGA datasets.
(A) The expression of class IIa HDACs in CRC tissues compared with normal tissues in the TCGA datasets (*, p <0.05). **(B)** HDAC4, HDAC5 and HDAC9 mRNA levels in indicated paired CRC tissues and adjacent normal tissues by qRT-PCR. Quantitative data are shown as mean +/− S.D. p-values for each indicated comparison are two-tailed paired and derived from Wilcoxon test. **(C)** Kaplan–Meier survival analysis of OS and DFS about class IIa HDACs (HDAC4, HDAC5, HDAC9) expression in CRC from the GEPIA database,cutoff%=70.

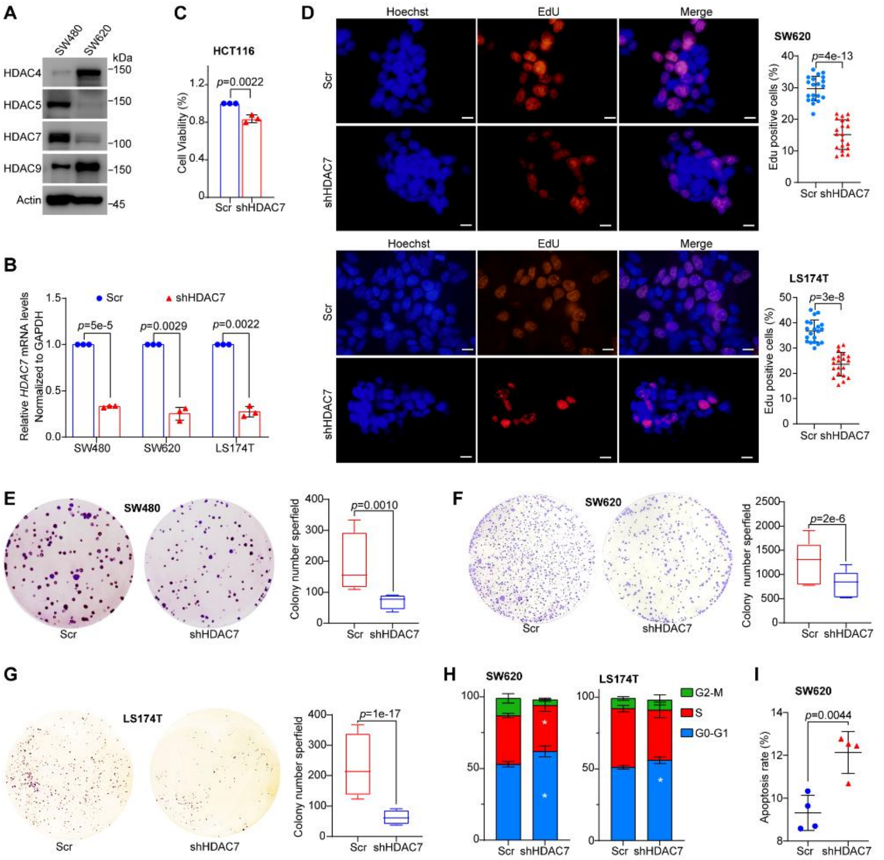

Figure S2-01. HDAC7 knockdown inhibits cell proliferation and survival in different CRC cell lines. (A) Western blot analysis of HDAC class IIa protein levels in SW480 and SW620 cells. **(B)** Relative HDAC7 mRNA levels in SW480, SW620 and LS174T cells measured by qRT-PCR and normalized to the GAPDH mRNA level following transfection of Scr (scramble shRNA) or shHDAC7. Quantitative data are mean +/− S.D. (n = 3; two-tailed unpaired Student's t-test). **(C)** HCT116 cell viability was assessed using the MTT assay in Scr and shHDAC7 knockdown. Quantitative data mean +/− S.D. (n=3; two-tailed unpaired Student's t-test). **(D)** Representative images and analysis of EdU incorporation assay in SW620 (top), LS174T (bottom) cells. EdU-positive cells (red) and Hoechst 33342–stained cells (blue). Scale bar, 100 µm. Quantitative data mean +/− S.D. (n=20; two-tailed unpaired Student's t-test without adjustment for multiple comparisons). **(E**, **F** and **G)**, Representative images show the ability of colony formation (left) in shHDAC7 and Scr SW480 (E), SW620 (F) and LS174T (G) cells. The number of colonies in each well was analyzed (right). Quantitative data are mean +/− S.D. p-values are derived from two-tailed unpaired Student's t-test without adjustment for multiple comparisons. **(H)** The cell cycle distribution of SW620 (left) and LS174T (right) was analyzed by flow cytometry. Bar charts show the percentages of cell cycle distribution. Quantitative data are mean +/− S.D. p-values for each indicated comparison. Two-tailed unpaired Student's t-test without adjustment for multiple comparisons (*, $p<0.05$). **(I)** SW620 cells were subjected to apoptosis determination by flow cytometry. The percentage of apoptotic cells was analyzed. Quantitative data are mean +/− S.D. (n = 4; two-tailed unpaired Student's t-test without adjustment for multiple comparisons).

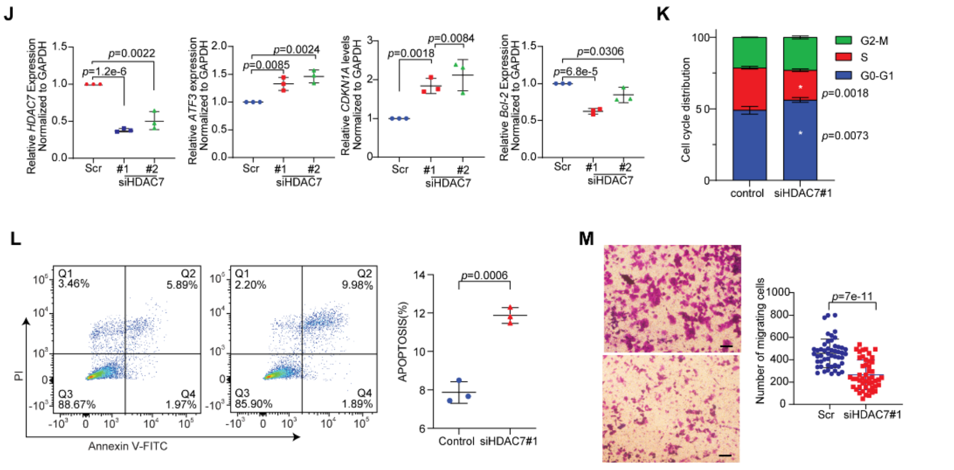

Figure S2-02. HDAC7 knockdown inhibits cell proliferation and survival in different CRC cell lines.
(J) Relative gene mRNA levels in SW480 cells measured by qRT-PCR and normalized to the GAPDH mRNA level following transfection of Scr (scramble siRNA) or siHDAC7 #1/#2. Quantitative data are shown as mean +/− S.D. (n = 3). p-values for each indicated comparison are two-tailed unpaired and derived from Student's t-test. **(K)** The cell cycle distribution of SW480 cells was analyzed in SW480 cells with HDAC7 knockdown(by siHDAC7 #1). Quantitative data are shown as mean +/− S.D. (n = minimum of three independent experiments for each condition). p-values for each indicated comparison are two-tailed unpaired and derived from Student's t-test. **(L)** Flow cytometry analysis showing knockdown of HDAC7 (by siHDAC7 #1) induced apoptosis in SW480 cells. Quantitative data are shown as mean +/− S.D. (n = 3). p-values for each indicated comparison are two-tailed unpaired and derived from Student's t-test without adjustment for multiple comparisons.(*, p<0.05). **(M)** Transwell assay showed that HDAC7 knockdown (by siHDAC7 #1) repressed SW480 cell migration. Quantified results are expressed as mean +/− S.D. (n = minimum of three independent experiments for each condition). p-values for each indicated comparison are two-tailed unpaired and derived from Student's t-test without adjustment for multiple comparisons. Migrated cells were stained purple with crystal violet. Scale bar: 100μm.

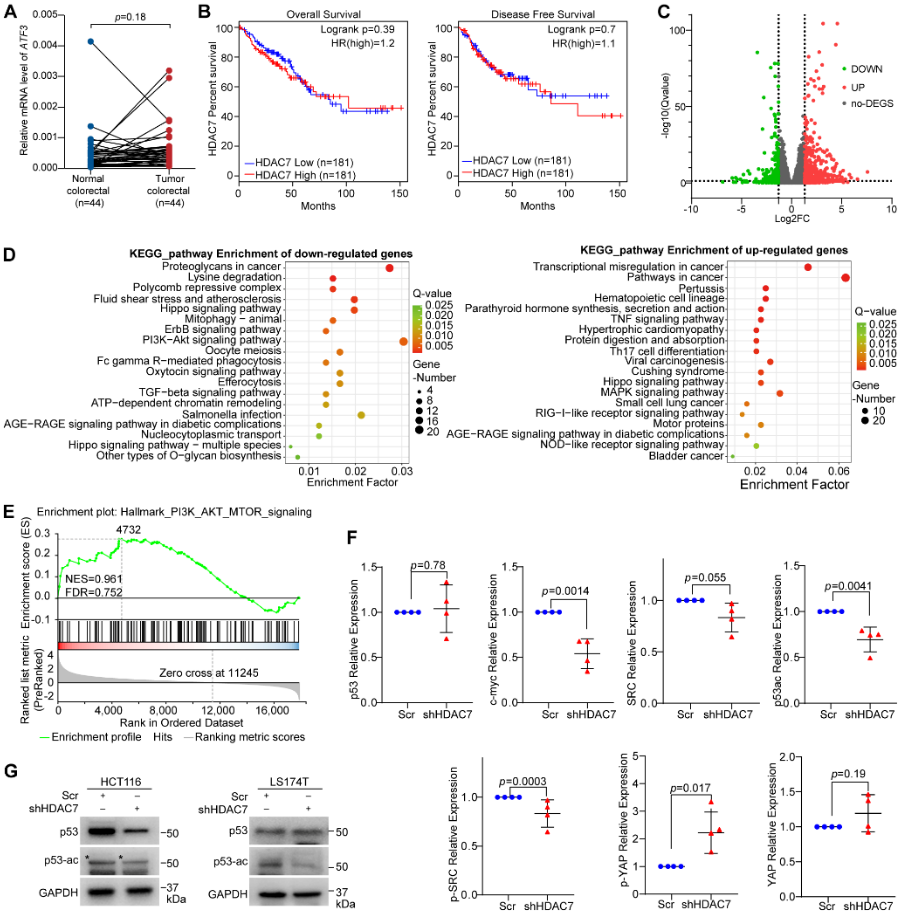

Figure S3. HDAC7 suppresses ATF3 expression and promotes cancer signaling pathways for tumor cell growth and survival. (A) ATF3 relative mRNA levels in indicated paired CRC tissues and adjacent normal tissues by qRT-PCR. Quantitative data are shown as mean +/− S.D. *p*-values for each indicated comparison are two-tailed paired and derived from Wilcoxon test. **(B)** Kaplan–Meier survival analysis of OS and DFS about HDAC7 expression in CRC from the GEPIA database, cutoff%=50. **(C)** RNA-seq defines HDAC7-dependent transcriptional programs. Volcano plot showing distribution of differentially expressed genes between a SW480 HDAC7 knockdown cells and Scr control cells. Adjusted P value<0.05. **(D)** KEGG pathway enrichment analysis differentially expressed genes in SW480 cells upon stable HDAC7-knockdown (shHDAC7) (|log2FC| ≥1, Q value ≤0.01). Down-regulated (left) and Up-regulated (right) (Bubble size represents gene count; color indicates enrichment significance. **(E)** The GSEA of PI3K/AKT pathway genes in SW480 cells compared with Scr. **(F)** Quantitative analysis of indicated proteins in the WB data (Fig 3i) with Scr control and shHDAC7 groups. The relative expression was normalized by GAPDH. Data are presented as mean +/− S.D. (n=3). p-values for each indicated comparison are two-tailed unpaired and derived from Student's t-test. **(G)** Western blot analysis of indicated proteins in Scr control and shHDAC7 knockdown of HCT116/LS174T cells. The relative expression was normalized by GAPDH.

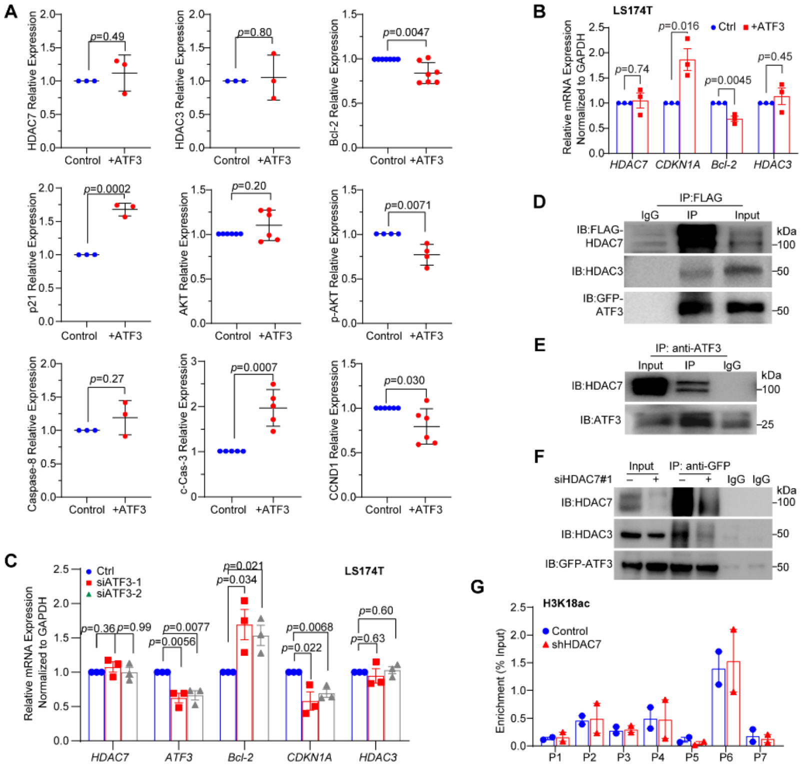

Supplementary Figure 4. HDAC7 modulates ATF3 functional alteration and its self-transcriptional regulation. (A) Quantitative analysis of Western blot of indicated proteins in control and over-ATF3 groups. The relative expression was normalized by GAPDH. Data are presented as mean +/− S.D. (n=3). p-values for each indicated comparison are two-tailed unpaired and derived from Student's t-test. **(B)** Relative *HDAC7*, *CDKN1A* (encodes p21), *Bcl-2* and *HDAC3* mRNA levels in LS174T cells by qRT-PCR after the overexpression of ATF3. Quantitative data are shown as mean +/− S.D. (n = minimum of three independent experiments for each condition). p-values for each indicated comparison are two-tailed unpaired and derived from Student's t-test. The relative expression was normalized by GAPDH. **(C)** Relative *ATF3*, *HDAC7*, *CDKN1A* (encodes p21), *Bcl-2* and *HDAC3* mRNA levels in LS174T cells by qRT-PCR following the transient knockdown of ATF3. Quantitative data are shown as mean +/− S.D. (n = minimum of three independent experiments for each condition). p-val- ues for each indicated comparison are two-tailed unpaired and derived from Student's t-test. The relative expression was normalized by GAPDH. **(D)** 293T cell lysates were subject to immunoprecipitation with control IgG, anti-FLAG. The co-IP were blotted with anti-GFP, anti-FLAG and anti-HDAC3 antibodies. **(E)** Co-immunoprecipitation analysis of association between endogenous HDAC7 and ATF3 in SW480 cells. Immunoblotting was carried out using anti-ATF3 and anti-HDAC7 antibodies. **(F)** Co-IP assay detecting the association of GFP-ATF3 with HDAC7 and HDAC3 treated with control or siRNA-HDAC7. GFP-ATF3 was transfected into 293T cells and then immunoprecipitated with anti-GFP, anti-HDAC7 and anti-HDAC3 antibodies. **(G)** shHDAC7 SW480 cells and Scr cells were harvest for ChIP analysis with H3K18ac, antibodies. The enrichment of H3K18ac at the ATF3 promoter or enhancer were analyzed using qRT-PCR. Values represent the average of two independent experiments with mean +/− S.E.M. (n =2).

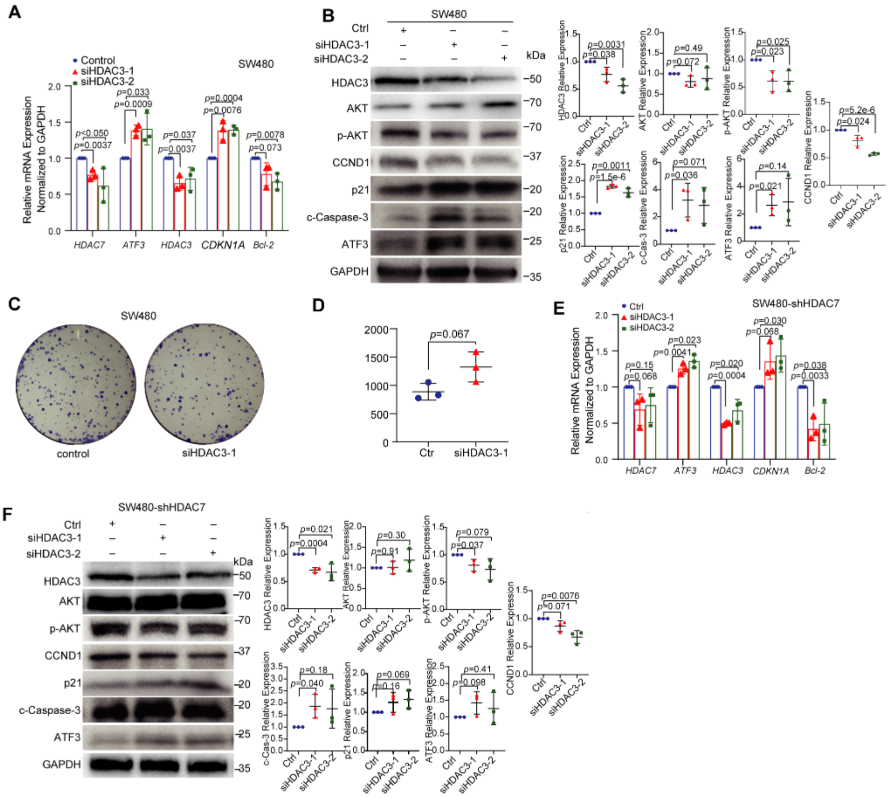

Supplementary Fig. 5 |HDAC3 modulates ATF3 functional alteration and its self-transcriptional regulation.(A) Relative ATF3,HDAC7, CDKN1A (encodes p21), Bcl-2 and HDAC3 mRNA levels in SW480 cells by qRT-PCR following the knockdown of HDAC3. Quantitative data are shown as mean +/− S.D. (n = minimum of three independent experiments for each condition). p-values for each indicated comparison are two-tailed unpaired and derived from Student's t-test. The relative expression was normalized by GAPDH.**(B)**Western blot analysis of key signaling proteins in control (Ctrl） and siRNA HDAC3-knockdown SW480 cells (normalized to GAPDH). And Quantitative analysis of Western blot of indicated proteins. The relative expression was normalized by GAPDH. Data are presented as mean +/− S.D. (n=3). p-values for each indicated comparison are two-tailed unpaired and derived from Student's t-test. **(C)**Colony-formation assay of SW480 cells treated with control (Ctrl） and siRNA HDAC3.**(D)**Quantitative data are mean ± S.D. (n ≥ 3; two-tailed unpaired and derived from Student's t-test). **(E)**Relative ATF3,HDAC7, CDKN1A (encodes p21), Bcl-2 and HDAC3 mRNA levels in shHDAC7 SW480 cells by qRT-PCR following the knockdown of HDAC3. Quantitative data are shown as mean +/− S.D. (n = minimum of three independent experiments for each condition). p-values for each indicated comparison are two-tailed unpaired and derived from Student's t-test. The relative expression was normalized by GAPDH.**(F)**Western blot analysis of key signaling proteins in control (Ctrl） and siRNA HDAC3-knockdown shHDAC7-SW480 cells (normalized to GAPDH). And Quantitative analysis of Western blot of indicated proteins. The relative expression was normalized by GAPDH. Data are presented as mean +/− S.D. (n=3). p-values for each indicated comparison are two-tailed unpaired and derived from Student's t-test.

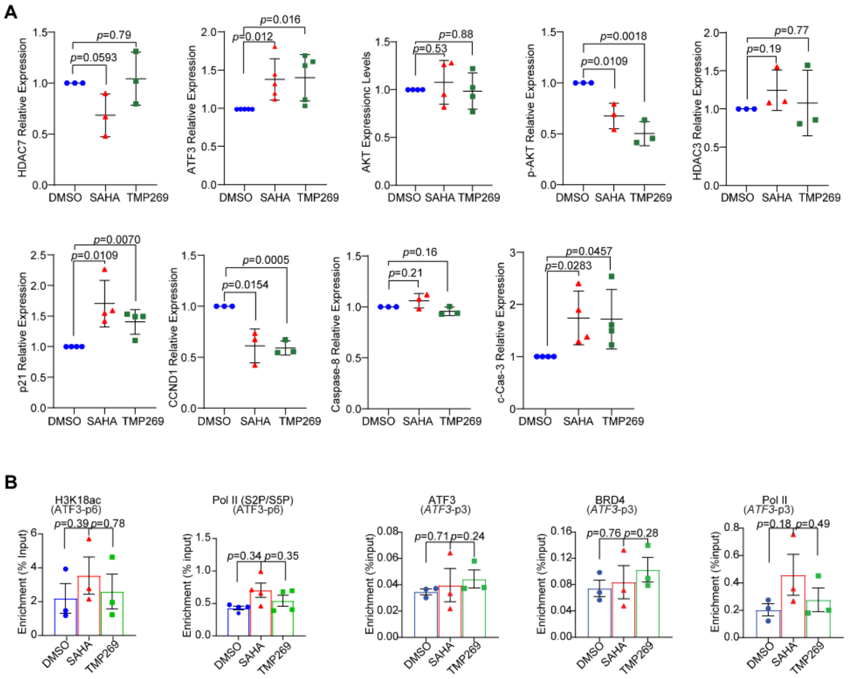

Figure S6. HDAC7 inhibition promotes ATF3 recruitment with transcription coactivators and activats ATF3 self-transcription. (A) Western blot analysis of indicated proteins following the treatment of 0.05%DMSO, SAHA (1.5 μM) or TMP269 (3 μM) in SW480 cells. The relative expression was normalized by GAPDH. Data are presented as mean +/− S.D. p-values for each indicated comparison are two-tailed unpaired and derived from Student's t-test. **(B)** ChIP-PCR analysis of H3K18ac, Pol II (S2P/S5P), ATF3, BRD4 and wild-type Pol II enrichments at ATF3 promoter or enhancer loci (p6, p3) in SW480 cells after addition of 0.05% DMSO, 1.5 μM SAHA or 3 μM TMP269. Values represent the average of mean +/− S.E.M (n = minimum of two independent experiments).

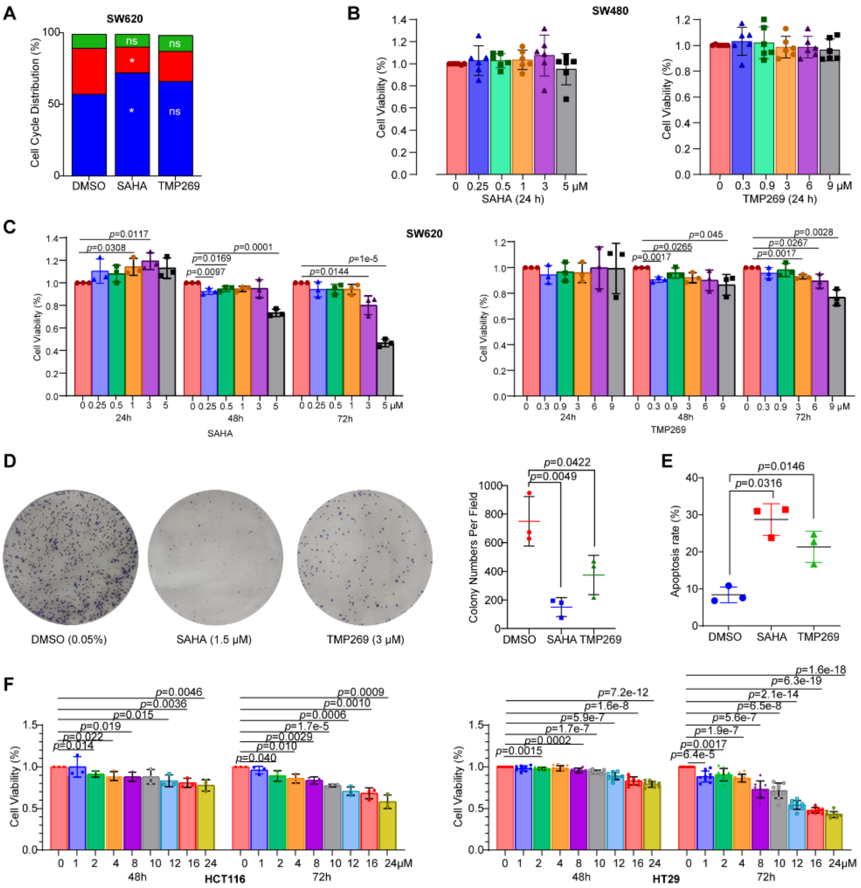

Figure S7. HDAC inhibiters suppress colon cancer cell proliferation and induce apoptosis. (A) Cell cycle distribution of SW620 cells treated with DMSO, 1.5 μM SAHA or 3 μM TMP269. Bar charts show the percentages of cell cycle distribution. Quantitative data are mean +/− S.D. (n=3; two-tailed unpaired and derived from Student's t-test without adjustment for multiple comparisons; *, $p < 0.05$). **(B)** Viability of SW480 cells treated with increasing concentrations of SAHA (0, 0.25, 0.5, 1, 3 and 5 μM) (left) or TMP269 (0, 0.3, 0.9, 3, 6 and 9 μM) (right) for 24 h (MTT assay). Quantitative data are mean +/− S.D. (n ≥ 3; two-tailed unpaired Student's t-test). **(C)** Time- and dose-dependent effects of SAHA (0, 0.25, 0.5, 1, 3 and 5 μM) (left) or TMP269 (0, 0.3, 0.9, 3, 6 and 9 μM) (right) for 24, 48 and 72h on SW620 cell viability. Quantitative data are mean +/− S.D. (n=3; two-tailed unpaired Student's t-test). **(D)** Colony-formation assay in SW480 cells treated with DMSO, 1.5 μM SAHA, or 3 μM TMP269. Representative images (left) and quantification (right) are shown. Quantitative data are mean +/− S.D. (n=3; two-tailed unpaired Student's t-test without adjustment for multiple comparisons). **(E)** Apoptosis analysis (Annexin V/PI staining) of SW620 cells treated with DMSO, 1.5 μM SAHA or 3 μM TMP269. Quantitative data mean +/− S.D. (n=3; two-tailed unpaired Student's t-test without adjustment for multiple comparisons). **(F)** Viability of HCT116 and HT29 cells treated with increasing concentrations of TMP269 (0, 1, 2, 4, 8,10,12,16 and 24 μM) for 48/72 h (MTT assay). Quantitative data are mean +/− S.D. (n ≥ 3; two-tailed unpaired Student's t-test).
